# Efficacy and Safety of Filgrastim and Its Biosimilars to Prevent Febrile Neutropenia in Cancer Patients: A Prospective Study and Meta-Analysis

**DOI:** 10.3390/biology10101069

**Published:** 2021-10-19

**Authors:** Shruti Rastogi, Vivekananda Kalaiselvan, Sher Ali, Ajaz Ahmad, Sameer Ahmad Guru, Maryam Sarwat

**Affiliations:** 1 Indian Pharmacopoeia Commission, Ministry of Health & Family Welfare, Government of India, Sector-23, Raj Nagar, Ghaziabad 201002, Uttar Pradesh, India; shruti.ipc@gov.in (S.R.); kalaiselvan.ipc@gov.in (V.K.); 2 Amity Institute of Pharmacy, Amity University, Noida 201301, Uttar Pradesh, India; 3 School of Basic Sciences and Research, Department of Life Sciences, Sharda University, Greater Noida 201310, Uttar Pradesh, India; sher.ali@sharda.ac.in; 4 Department of Clinical Pharmacy, College of Pharmacy, King Saud University, Riyadh 11451, Saudi Arabia; aajaz@ksu.edu.sa; 5 Lurie Children’s Hospital, Department of Pediatric Surgery, Northwestern University, Chicago, IL 60611, USA; sguru@luriechildrens.org

**Keywords:** filgrastim, pegfilgrastim, meta-analysis, granulocyte-colony stimulating factors, febrile neutropenia, systematic literature review, biosimilars

## Abstract

**Simple Summary:**

Febrile neutropenia is the serious side-effect associated with myelosuppressive chemotherapy. Filgrastim, the first granulocyte colony-stimulating factor (G-CSF) was approved by the Food and Drug Administration for the treatment of neutropenia. Subsequently, pegfilgrastim (long-acting G-CSF) and filgrastim biosimilars were developed to have comparable efficacy to filgrastim. Therefore, it is necessary to produce a systematic review and meta-analysis that provides evidence that filgrastim is more efficacious than placebo/no-treatment, as it provides evidence on the comparable efficacy of filgrastim versus pegfilgrastim and biosimilar filgrastim.

**Abstract:**

**Background**: The aim of this review and meta-analysis was to identify, assess, meta-analyze and summarize the comparative effectiveness and safety of filgrastim in head-to-head trials with placebo/no treatment, pegfilgrastim (and biosimilar filgrastim to update advances in the field. **Methods**: The preferred reporting items for systematic reviews and meta-analyses PRISMA statement were applied, and a random-effect model was used. Primary endpoints were the rate and duration of grade 3 or 4 neutropenia, and an incidence rate of febrile neutropenia. Secondary endpoints were time to absolute neutrophil count ANC recovery, depth of ANC nadir (lowest ANC), neutropenia-related hospitalization and other neutropenia-related complications. For filgrastim versus biosimilar filgrastim comparison, the primary efficacy endpoint was the mean difference in duration of severe neutropenia DSN. **Results**: A total of 56 studies were considered that included data from 13,058 cancer patients. The risk of febrile neutropenia in filgrastim versus placebo/no treatment was not statistically different. The risk ratio for febrile neutropenia was 0.58, a 42% reduction in favor of filgrastim. The most reported adverse event with FIL was bone pain. For pegfilgrastim versus filgrastim, no statistically significant difference was noted. The risk ratio was 0.90 (95% CI 0.67 to 1.12). The overall difference in duration of severe neutropenia between filgrastim and biosimilar filgrastim was not statistically significant. The risk ratio was 1.03 (95% CI 0.93 to 1.13). **Conclusions**: Filgrastim was effective and safe in reducing febrile neutropenia and related complications, compared to placebo/no treatment. No notable differences were found between pegfilgrastim and filgrastim in terms of efficacy and safety. However, a similar efficacy profile was observed with FIL and its biosimilars.

## 1. Introduction

Neutropenia and its complications, including febrile neutropenia (FN) and infections, are the major dose-limiting toxicity associated with myelosuppressive chemotherapy (MSC). This is characterized by a significant reduction in neutrophilic count, accompanied by fever. Severe neutropenia (grade 3 and grade 4) is characterized with a neutrophil count of <1.0 × 10^9^/L and <0.5 × 10^9^/L, respectively [1]. FN complications are considered a medical emergency and require prolonged hospitalization [2]. These patients then need to be administered with broad-spectrum antibiotics, which indirectly increase the treatment cost to the patients [1]. In particular, FN risk leads to chemotherapy delay, unplanned dose reduction, substantial morbidity and mortality. Studies have shown that reduction in intensity of chemotherapy are associated with poor outcomes, causing a detrimental effect on the quality of life [3,4].

Granulocyte colony-stimulating factors (G-CSF) are glycoproteins that stimulate the proliferation and differentiation of neutrophil progenitor cells and the release of neutrophils from bone marrow into the blood [5,6]. G-CSFs are known to reduce the duration and severity of neutropenia and decrease the incidence of FN [7,8,9]. The current treatment guidelines for North America and Europe, which include National Comprehensive Cancer Network (NCCN) [10], Spanish Society of Medical Oncology (SEOM) [11], American Society of Clinical Oncology (ASCO) [12], Infectious Diseases Society of America (IDSA) [12] and the European Society for Medical Oncology (ESMO) [13]. These bodies recommend the primary prophylaxis (PP) with G-CSF when the likelihood of developing FN is >20%. The G-CSF must be indicated based on patient characteristics, such as age >65 years, liver or kidney dysfunction, persistent neutropenia, or prior episodes of FN [11,13,14]. The prophylactic use of G-CSF is associated with reduction in incidence, severity and duration of FN and severe chronic neutropenia (SCN), reduction in hospitalization and lower mortality rate due to infection [1,15].

Filgrastim (FIL; Neupogen^®^) was the first G-CSF drug approved for treatment of neutropenia by the United States Food and Drug Administration (US FDA) in 1991 [16]. As of 2018, Neupogen^®^ is approved by the U.S. FDA mainly for 6 indications [17]. These include (1) decreasing the incidence of infection and prevent FN in patients with cancer receiving MSC; (2) reducing the time to neutrophil recovery and the duration of fever in patients with acute myeloid leukemia (AML); (3) reducing the duration of neutropenia and neutropenia-related clinical sequelae in patients with non-myeloid malignancies undergoing myeloablative chemotherapy followed by bone marrow transplantation (BMT); (4) likewise, mobilizing autologous hematopoietic progenitor cells into the peripheral blood for collection by leukapheresis (PBCL); (5) reducing incidence and duration of sequelae of severe neutropenia in symptomatic patients with SCN; (6) increasing survival of patients acutely exposed to myelosuppressive doses of radiation (hematopoietic syndrome of acute radiation syndrome) (HSARS). Other short-acting G-CSFs include lenograstim and tbo-filgrastim. However, because of the relatively short half-life of these short-acting G-CSFs, daily injections are required to stimulate neutrophil recovery. Subsequently, long-acting G-CSF drugs (pegfilgrastim (PEG-F) and lipegfilgrastim) were developed by increasing the molecular size and thus, evading renal clearance. These long-acting G-CSFs have decreased elimination and increased half-life in serum after subcutaneous injection [18]. Previously randomized controlled trials (RCTs) and observational studies have suggested that a single dose of PEG-F is equivalent and in some instances superior to 10–14 days of daily course of FIL [19,20].

In last few years, with FIL patent expiry, several biosimilar filgrastim (Bio-F) have been developed; these include Zarxio^®^, Granix^®^ and Nivestym^®^ [21]. These Bio-F were developed to have similar analytical comparability and clinically no difference in quality, safety and efficacy to their reference medicine [22]. The pathways of Bio-F were set by the European Medicine Agency (EMA) and U.S. FDA, mainly checking comparability on the basis of pharmacokinetic/pharmacodynamics properties, and preclinical and physicochemical characteristics based on one indication; they rely on the extrapolation of data of innovators to obtain approvals in other indications [23]. Several studies have shown that PP with FIL versus placebo control treatment/no-treatment (PCT/NT) in cancer patients is associated with reduction in intensity of infection and overall mortality rate [24,25]. Studies have also shown that PEG-F is more effective than FIL for chemotherapy-induced neutropenia [26,27,28,29]. A real-world comparative efficacy study of PP with FIL, PEG-F and Bio-F found no difference in terms of efficacy between FIL and Bio-F. In addition, risks of FN and FN-related complications were generally lower for prophylaxis with PEG-F than with short-acting G-CSF [30]. Several trials have evaluated the efficacy of FIL versus PEG-F in clinical and real-world settings. However, only a single review has evaluated the efficacy of FIL versus PCT/NT in all its U.S. approved indications [31].

Thus, a descriptive study summarizing CTs and real-world data for evaluating FIL versus PEG-F and Bio-F across its indications can be useful to the researchers. Our decision to study the effectiveness profile of addressed G-CSFs depended only on the current availability of homogeneous series of published CTs that have evaluated the prophylactic use of FIL in its approved indications.

## 2. Methods

These analyses were conducted in accordance with the Preferred Reporting Items for Systematic Reviews and Meta-analysis (PRISMA).

### 2.1. Search Strategy

A systematic search of PubMed, Cochrane Central Register of Controlled Trials, Cochrane Database of Systemic Reviews, Scholar, ClinicalTrials.gov, reference list of relevant articles from inception to July 2020 were searched. A previous systematic literature review by Dale et al. [31] presented a meta-analysis of originator FIL versus PCT/NT in its U.S. approved indications up to December 2015. Therefore, databases were searched from January 2016 onwards, whereas studies published prior to 2016 were identified from the existing review. For FIL versus PEG-F and Bio-F studies, a complete search was made since its inception. Bibliographies of retrieved papers, including product label information, were searched for any additional relevant studies.

Search terms included “Neupogen”, “filgrastim”, “granulocyte colony-stimulating factor”, “G-CSF/GCSF”, “recombinant human granulocyte colony stimulating factor (rhG-CSF/rhGCSF)”, “r-met-HUG-CSF/r-metHuG-CSF, “hematopoietic growth factor” in combination with the keywords “pegfilgrastim”, “Neulasta”, and “filgrastim biosimilars”. The full search strategy is available in Supplementary Materials Table S1.

### 2.2. Inclusion and Exclusion Criteria

To be eligible for inclusion, we considered comparative studies, including RCTs (patients randomized to FIL or its comparators, non-randomized controlled trials (NRCTs) (patients not randomly assigned to FIL or comparators) or observational studies (longitudinal studies, open-label studies, registry studies), evaluating the efficacy and safety of originator FIL with other short- or long-acting G-CSF, including Bio-F in its U.S. approved indications. There were no restrictions regarding age, gender, duration of the study or geographical region. Based on the title, abstracts and/or full manuscript, the study selection, data extraction and review and quality assessment were done by two independent reviewers (MS, VK). Areas of disagreement or uncertainty were resolved by consensus. For consistency with the existing systematic review [31], the studies with <50 patients receiving FIL for all the indications, except for SCN and PBSCL (for SCN ≥ 10 patients and for PBSCL ≥ 40 were included), were excluded. The study excluded the following: non-comparative studies evaluating the efficacy and safety of biosimilars (comments, editorials, case reports, non-research letters, and narrative reviews); economic analyses; cancer registry reports; workshops; studies in animals but not in humans; single-arm studies; and results published in languages other than English. The quality of the randomized studies was assessed by the Cochrane risk-of-bias tool version 2 (RoB 2) [32] as recommended by the Cochrane Collaboration.

G-CSF reference medicines included in the meta-analysis were FIL, PEG-F and Bio-F. Other GCSFs, such as mecapegfilgrastim and empegfilgrastim, were not included for the calculation of FN incidence. The Bio-F investigated were Zarzio^®^ (also known as EP2006), tbo-filgrastim (also known as XM02), Fiprima^®^ and Nivestim^®^ (Hospira filgrastim).

### 2.3. Outcome Measures

The primary outcome of interest for efficacy and effectiveness were rate and duration of grade 3 or 4 neutropenia and incidence rate of FN. Secondary endpoints were time to absolute neutrophil count (ANC) recovery; depth of ANC nadir (lowest ANC); time to neutrophil recovery (defined as the time from chemotherapy administration until the ANC increased to ≥2.0 × 10^9^/L after the expected nadir); chemotherapy dose reductions/delays, reduced dose intensity and number of patients receiving full dose on schedule; incidence and duration of hospitalization and fever; and need for antibiotic prophylaxis or treatment. For FIL innovator and Bio-F comparison, the primary endpoint of efficacy was the duration of FN (DSN) during cycle 1, and the secondary endpoint included the depth of ANC nadir (lowest ANC), time to neutrophil recovery, and FN.

For safety outcomes, the incidence of adverse events (AEs) related to FIL, including muscle pain, bone pain, anemia, diarrhea, leukocytosis, thrombocytopenia, allergic reactions, alopecia, and anorexia and the incidence of serious adverse event (SAE) were collected. Moreover, on comparison of FIL with PCT/NT, any AEs experienced were associated with FIL. In the case of FIL innovator and Bio-F comparison, the safety endpoints were muscle pain, bone pain and SAEs recorded across all CT cycles.

### 2.4. Data Abstraction and Analysis

The key characteristics of each study were extracted by the reviewers from full text versions of the studies. Descriptive analyses were used to summarize the studies and included the first author’s name and year of publication, study design, cancer type and stage, country, patient characteristics (number of patients enrolled, age and sex), chemotherapy regimens and dose treatment. All data were summarized in a structured table to ensure consistency. The studies were classified, according to the filgrastim U.S. FDA approved indications.

Random-effect meta-analysis was used to synthesize the results from direct (head-to-head) trials for FN, grade 3 to 4 neutropenia and bone pain in CIN to compare the results from trials for FIL versus PCT/NT and FIL versus PEG-F; relative risks (RR) were determined. Heterogeneity across studies was assessed, using forest plots. The inconsistency index (I^2^) was used to quantify the percentage of variability due to heterogeneity rather than sampling error.

Meta-analysis methods for continuous outcome measures varied, due to differences in the availability of the reported results. For FIL and Bio-F comparison, mean treatment differences were extracted from each individual study. In the case that the median was provided instead of the mean, we assumed normality and considered it equal to mean. Where range was provided, we calculated SD as range/4. Due to limited and heterogeneous data, for other outcomes, the mean and median were summarized, and the meta-analysis was not performed.

The presence of publication bias for small study effect appraisal was assessed by visual examination of funnel plots. Statistical significance is based on data provided in original publication using *p* < 0.05. Computations were performed using MedCalc Version 19.6.1 software.

## 3. Results

**Figure 1:**
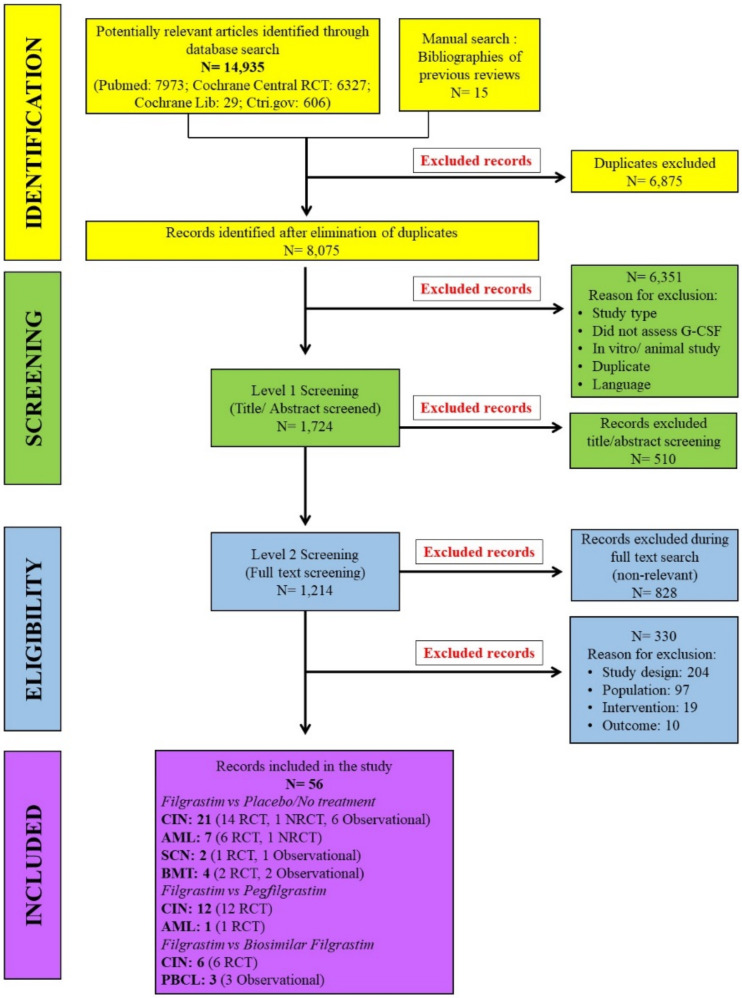
Study selection diagram. The flow diagram explains the selection process of clinical trials included in the meta-analysis. AML = acute myeloid leukemia; BMT = bone marrow transplantation; CIN = chemotherapy-induced neutropenia; NRCT = non- randomized controlled trial; PBCL = peripheral blood for collection by leukapheresis; RCT = randomized controlled trial; SCN = severe chronic neutropenia.

According to our research query, 14,950 potentially relevant records were identified. For FIL versus PCT/NT, studies published from 2016 onwards were identified from the literature search, and studies published prior to 2016 were identified from the previous review [31]. In addition, studies related to FIL versus PEG-F and FIL versus Bio-F were searched separately. After removal of duplicates, a total of 56 studies that satisfied the inclusion criteria were included in our systematic review. The PRISMA flow diagram describing the search strategy and study selection process is available in Figure 1.

### 3.1. Description of Studies

The studies represent data from 13,058 patients globally with FIL administered to 6072 patients, including 3998 patients from FIL-CIN (filgrastim in chemotherapy induced neutropenia patients) (FIL versus PCT/NT; FIL versus PEG-F; FIL versus Bio-F), 1069 patients from FIL-AML (filgrastim in acute myeloid leukemia patients) (FIL versus PCT/NT; FIL versus PEG-F), 79 patients from FIL-SCN (filgrastim in severe chronic neutropenia patients) (FIL versus PCT/NT), 671 patients from FIL-BMT (filgrastim in bone marrow transplantation) (FIL versus PCT/NT) and 255 patients from FIL-PBCL (filgrastim in peripheral blood for collection by leukapheresis) (FIL versus Bio-F).

Among the 56 studies included in the meta-analysis for approved indications, the following were included: 34 studies of FIL versus PCT/NT (Supplementary Materials Table S8) comprising 21 studies in CIN indication (14 RCTs [7,33,34,35,36,37,38,39,40,41,42,43,44,45], 1 NRCT [46] and 6 observational [47,48,49,50,51,52]); 7 studies in AML indication (6 RCTs [53,54,55,56,57,58] and 1 NRCT [59]); 2 studies in SCN indication (1 RCT [60] and 1 observational [61]); 4 studies in BMT indication (2 RCTs [62,63] and 2 observational [64,65]). A total of 13 studies of FIL versus PEG-F were included (Supplementary Materials Table S9): 12 RCTs of CIN indication [8,9,19,66,67,68,69,70,71,72,73,74] and 1 RCT of AML indication [75]. A total of 9 studies of FIL versus Bio-F were included (Supplementary Materials Table S10): 6 RCTs of CIN indication [76,77,78,79,80,81] and 3 observational studies of PBCL indication [82,83,84]. No studies evaluating FIL versus PCT/NT in HSARS indication met the eligibility criteria for data extraction.

Among the included studies, 17 reports provided data from the trials of breast cancer [8,9,19,39,47,66,67,68,69,70,71,72,76,77,78,80,81]; 1 study involved non-small-cell lung carcinoma (NSCLC) [46]; 4 studies were for small cell lung carcinoma (SCLC) [7,33,36,45]; 1 was for colorectal cancer [48]; 9 involved non-Hodgkin’s lymphoma (NHL) [34,35,42,63,64,70,73,74,79]; 7 studies involved acute leukemia lymphoma (ALL) [38,40,43,44,52,62,82]; 8 AML [53,54,55,56,57,58,59,75]; 2 SCN [60,61]; 1 with germ cell tumors [37]; 1 with metastatic neuroblastoma [41]; and 6 were multiple myeloma (MM) [49,50,51,65,83,84]. Across trials, the percentage of females ranged from 31% to 100% in the case of breast cancer.

Full descriptions of chemotherapy regimens and treatments are provided in Table 1, Supplementary Materials Tables S2–S4 and Supplementary Materials Tables S11–S13. Baseline population characteristics are summarized in Supplementary Materials Tables S5–S7. The assessment of quality of each randomized trial is shown in Supplementary Materials Figure S1. Publication bias among studies is shown by funnel plots in Supplementary Materials Figures S2 and S3.

### 3.2. Outcome of FIL versus PCT/NT by Indication

#### 3.2.1. CIN Indication

**Figure 2:**
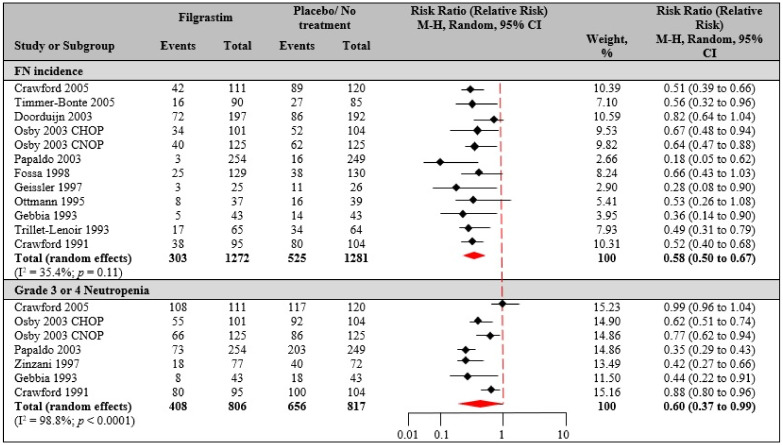
Filgrastim versus placebo/no treatment. Risk estimates in CIN patients for FN incidence (2553 total patients; filgrastim, *n* = 1272; placebo or no treatment, *n* = 1281) and grade 3 or 4 neutropenia incidence (1623 total patients; filgrastim, *n* = 806; placebo or no treatment, *n* = 817). Random effects meta-analysis was performed for the outcomes to compare data from clinical trials for filgrastim versus placebo or no treatment, and the relative risk was determined.

FN incidence: 11 of 21 studies [7,33,34,35,36,37,39,40,45,51,52] mentioned FN incidence and were selected for study. The studies enrolled 2553 patients in total (FIL, *n* = 1272; PCT/NT, *n* = 1281). All studies used the same definition of FN (ANC <0.5 × 10^9^/L and temperatures ≥38.2 °C), except for 4 RCTs [7,45,51,52], which defined FN as ANC <1 × 10^9^/L and ≥38.2 °C. The risk of FN in patients receiving FIL ranged from 1% to 38% with a mean of 23% (95% CI: 16–30%), compared to PCT/NT from 7% to 74% with a mean of 42% (95% CI: 32–52%). The PP with FIL decreased the risk of FN, compared with PCT/NT (RR 0.58, 95% CI 0.50–0.67) (Figure 2). Statistical heterogeneity was observed in the analyses as shown by I^2^ statistic and Q-statistic (I^2^ = 35.4%, Q-value = 17, *p* = 0.11), using the random effect model. The variation is influenced by factors such as cancer type, patient age, chemotherapy regimen, number of cycles and cycle length.

Grade 3 or 4 neutropenia: A total of 6 of 21 studies [7,33,35,39,42,51] reported grade 3 or 4 neutropenia incidence and were included in the meta-analysis. Data analyzed were from 1576 total patients (FIL, *n* = 408; PCT/NT, *n* = 656). The incidence of developing grade 3 or 4 neutropenia was significantly lower with FIL, compared to PCT/NT (RR 0.60, 95% CI 0.37 to 0.99). The Q-statistic was statistically significant, suggesting evidence of relatively high level of statistical heterogeneity in the combined studies (I^2^ = 98.8%, Q-value = 507.1, *p* < 0.0001).

Duration of grade 3 or 4 neutropenia: Three RCTs [7,38,45] and one NCT [46] reported duration of grade 3 or 4 neutropenia. In all the 4 studies, the median duration was shorter for FIL versus PCT/NT, and ranged from 3 to 13 days.

Documented infection: Of 11 trials, 8 RCTs [7,34,37,38,42,43,45,50] and 3 observational studies [40,51,52] documented infection. The risk of documented infection in the FIL group ranged from 1.5% to 78% with a mean of 21.2 (95% CI 7.4 to 35). The risk of infections in PCT/NT ranged from 1% to 77% with a mean of 33.2 (95% CI 16.3 to 50.1). Three RCTs [34,42,43] reported significantly lower infection rates with FIL compared to PCT/NT, whereas two RCTs [38,45] and two observational studies [50,52] reported non-significant results.

Hospitalization: Seven RCTs [7,34,35,36,38,41,43], one NRCT [46] and one observational study [50] reported hospitalization with statistical comparison provided in six RCTs [7,34,35,38,43,45] and one observational study [50]. The mean hospitalization days for FIL were 10 days, compared to PCT/NT 14 days. In NRCT [46], the mean days of hospitalization with FIL were lower in NSCLC but higher in NHL. Significant improvement in hospital outcomes were observed in the granulocytopenic fever requiring hospitalization study [35], infection-related hospitalization, and median days of hospitalization in two studies [38,43]. There was no significant difference in hospitalization observed in the observational study [50] with FIL PP versus no FIL PP.

Relative dose intensity (RDI), dose reduction and treatment delay: Five RCTs reported RDI with statistical comparison provided in all studies. In studies with aggressive NHL [35] and germ cell tumor [37], RDI was significantly improved with FIL versus PCT/NT but not significantly different in breast cancer [39] and NHL [34,42] studies. In observational studies, significant improvement in RDI was observed in the multiple carcinoma study [51] in patients treated with FIL. In another observational study, [47] patients who received FIL (98%; range of 75%–117%) received a significantly higher mean RDI of their chemotherapy than those who were not given FIL (95%; range: 60–100%; *p* = 0.005). Chemotherapy dose reduction and dose delay was observed in two RCT [39,45], one NRCT [46] and one observational study [47] with statistical comparison provided in two studies. In RCTs, the dose reduction and dose delay were significantly reduced with FIL, compared to PCT/NT, in breast cancer [39]. However, in SCLC [45], significant dose reduction was observed but statistical significance was not mentioned for dose delay. In the observational study [47], both dose reduction and delay were not significant; however, in NRCT [46], the dose reduction was reduced but statistical significance was not reported. These were some of the avoidable omissions that made the study insipid.

Overall Survival (OS): There were generally fewer OS events among patients receiving FIL versus PCT/NT in the PCT studies. Only 8 trials reported OS [7,34,35,37,38,39,42,45]. The median follow-up time were 30 months [42], 33 months [34], 55 months [39], 57 months [35] and 4.7 years [38]. Two studies [34,38] reported no significant results, whereas in other studies, no statistical comparison was shown. None of the studies reported a difference in the survival rates of FIL versus PCT/NT.

Adverse Events (AEs): The results analyzed from 4 of 21 studies [7,38,44,45] reported sufficient data on bone pain. The mean frequency of bone pain among control subjects was 8.6% (95% CI: 1.5% to 16%; range, 1% to 24%), which was less than that in patients receiving FIL with mean 15% (95% CI: 5% to 25%; range, 1% to 35%). The RR of developing bone pain was higher with FIL versus PCT/NT (RR 1.75, 95% CI 0.94 to 3.28). Musculoskeletal pain was reported in an additional six studies ranging from 1.0% to 22% [35,42,44,45,51,52]. Other AEs reported were fatigue, nausea, headache, alopecia, thrombocytopenia and oral, cardiac, liver and general toxicities.

#### 3.2.2. AML Indication

Seven studies [53,54,55,56,57,58,59] evaluating FIL versus PCT/NT in AML indication enrolled 2075 patients in total, with FIL administered to 1028 patients. Four RCTs [53,54,56,58] reported the effect of FIL following induction chemotherapy, one RCT [55] reported the effect of FIL on outcomes following both induction and consolidation chemotherapy, and one RCT [57] and one NRCT [59] reported the effect of FIL following consolidation chemotherapy.

Duration of Grade 3 or 4 neutropenia: Two RCTs [57,58] reported grade 3 or 4 neutropenia. In both the studies, the median duration of neutropenia was shorter with FIL compared to PCT/NT and ranged from 10 to 12 days.

Documented infection: Five trials in AML indication, four RCTs [53,56,57,58] and one NRCT [59] documented infection. The mean risk of documented infection in the FIL group was 51 (95% CI 30.5 to 71.5), compared with a mean risk in PCT/NT group of 52.2 (95% CI 28.4 to 76) without significant heterogeneity.

Hospitalization: Four RCTs [54,56,57,58] and one NRCT [59] reported hospitalization with statistically significant results in two RCTs [58]. The mean hospitalization days were 27 days (95% CI: 24% to 30%) compared to the placebo of 33 days (95% CI: 27% to 39%). In AML patients, 4 RCTs reported OS receiving induction therapy, with no statistical difference observed between FIL versus PCT/NT in 3 RCTs [53,54,58]. The median follow-up time was 20 months [53] and 7 years [58].

AEs: Two of seven studies in AML indication [56,75] reported enough data on bone pain. The AEs reports were the same as previously discussed in the CIN indication. In addition, the AEs reported in AML indication included skin rash. A study [53] in AML indication reported Sweet’s disease, chest pain, generalized pruritus and skin rash in FIL patients.

#### 3.2.3. SCN Indication

Two studies [60,61] evaluated SCN indication and enrolled 169 patients with FIL administered to 79 patients. Among them, one was observational [61] and the other was a RCT phase III study [60].

#### 3.2.4. BMT Indication

Two RCTs [62,63] and two observational studies [64,65] that assessed FIL on haemopoietic recovery following high dose chemotherapy with bone marrow transplantation or PBSC support enrolled 922 patients in total, with FIL administered to 671 patients.

One RCT [63] compared two different doses of filgrastim 5 µg/kg/dose, s.c. bolus versus 10 µg/kg/dose continuous infusion following autologous BMT in patients with Hodgkin’s disease and NHL. The results showed no difference in the median time to reach ANC and in the median duration of neutropenia. The incidence and duration of AEs were also the same in both groups, concluding that the recommended dose of FIL after BMT should be 5 µg/kg/dose. Another RCT [62] enrolled pediatric patients with FIL administered to 51 patients suffering from hematological malignancies and solid tumors who underwent autologous PBPC transplantation. The median time to achieve ANC >0.5 × 10^9^/L was 10 days (range: 7–14) with FIL and 11 days (range: 8–21) in control group (*p* < 0.009). The median time to platelet >20 × 10^9^/L was 12 days in both groups (*p* = nonsignificant). The median time to platelet >50 × 10^9^/L was 15 days with FIL and 14 days in the control group (*p* < 0.005). In patients who received <5 × 10^6^/kg CD34 + cells, the median time to platelets >20 × 10^9^/L and >50 × 10^9^/L was similar with or without FIL (12 and 15 days, respectively).

The observational study [64] compared FIL-mobilized PBSC transplantation with FIL-primed autologous BMT. Patients affected by NHL or HL were selected for autologous transplantation. The results showed that the median time to platelet recovery >20 × 10^9^/L was 13 days for BM and 11 days for peripheral blood. The median time of hospital stay after reinfusion was non-significantly less for primed peripheral blood as compared to primed BMT (15.5 days versus 16.5 days). Thus, the results were similar for FIL-primed peripheral blood and FIL-primed BMT with an advantage of only 1 day in neutrophil recovery and 1 day in hospitalization stay. The other observational study [65] is a cohort one, performed on autologous SCT for MM with or without administering growth factors. The results suggested that it was feasible to perform autologous SCT without growth factors.

### 3.3. Outcome of FIL versus PEG-F by Indication

#### 3.3.1. CIN Indication

**Figure 3:**
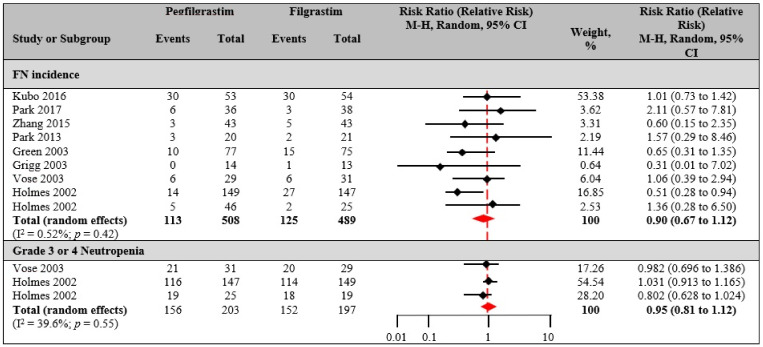
Pegfilgrastim versus filgrastim. Risk estimates in CIN patients for FN incidence (997 total patients; pegfilgrastim, *n* = 508; filgrastim, *n* = 489) and grade 3 or 4 neutropenia incidence (400 total patients; pegfilgrastim, *n* = 203; filgrastim, *n* = 197). Random effects meta-analysis was performed for the outcomes to compare data from clinical trials for pegfilgrastim versus filgrastim, and the relative risk was determined.

FN incidence: In FIL versus PEG-F head-to-head trials, the pooled comparison suggested that PEG-F had a lower risk of FN than those who received FIL (Figure 3). The overall RR was 0.90 (95% CI 0.67 to 1.12). Statistical heterogeneity as shown by the I^2^ statistic and Q statistic was I^2^ = 0.52%, Q-value = 11.7, and *p* = 0.42, using the random effect model. Among 12 studies that reported FN incidence, the risk of FN was statistically lower among patients treated with PEG-F in 1 study [9], numerically lower in 3 studies [8,68,71] and numerically higher in 8 studies [19,66,67,69,70,72,73,74]. FN-related hospitalization was less commonly reported in the included studies. One study [66] that provided statistical comparisons of FN-related hospitalization among patients treated with PEG-F versus FIL non-significantly reported lower risks of FN-related hospitalization among patients treated with PEG-F.

Grade 3 or 4 neutropenia: Three of twelve studies [9,19,74] reported grade 3 or 4 neutropenia incidence and were included in the meta-analysis. Data analyzed were from 400 total patients (PEG-F, *n* = 156; FIL, *n* = 152). The differences between the arms were quite small (RR 0.95, 95% CI 0.81 to 1.12). The Q statistic was statistically insignificant (I^2^ = 39.6%, Q-value = 3.31, *p* = 0.55). There was no appreciable difference in the duration of grade 3 or 4 neutropenia in studies with a mean of 1.9 (95% CI 1.29 to 2.5).

Time to ANC recovery: Five studies [8,9,19,67,68] compared the efficacy of FIL versus PEG-F, with one study [19] reflecting shorter time to recovery for PEG-F, three studies [9,66,68] estimating in the opposite direction and the remaining one study [8] showing the same time in ANC recovery.

AEs: Data analyzed from 4 of 12 studies [19,68,70,71] reported sufficient data on bone pain. The mean frequency of bone pain among PEG-F group was 3.1% (95% CI: 0.4% to 5.8%; range, 1% to 7%), which was less than among patients receiving FIL with mean 6.7% (95% CI: 5.0% to 8.4%; range, 5% to 9%). The RR of developing bone pain was non-significantly higher with FIL, compared to PEG-F (RR 0.56, 95% CI 0.26 to 1.19). Other AEs experienced were back pain, arthralgia, myalgia, thrombocytopenia, and general toxicities.

#### 3.3.2. AML Indication

The multicenter phase II RCT [75] analyzed patients with AML and compared single dose of PEG-F versus daily dose of FIL in 83 patients (Peg-F = 42 patients versus FIL = 41 patients). Median time to ANC recovery was 22.0 days (difference between groups 0.0; 95% CI: 0.9 to 1.9 days) in both groups during induction 1. During consolidation, recovery occurred after a median of 17.0 days for PEG-F versus 16.5 days for FIL (difference 0.5 days; 95% CI: 1.1 to 2.1). The FN incidence was 81% in PEG-F (median duration 15 (11, 20) days) versus 88% in FIL group (median duration 14 (11.5, 18.5) days) during induction 1. During consolidation, fever was reported in more patients in the PEG-F group (77%) versus the FIL group (58%), but the median duration was two in both groups. The AE profile was similar in both groups. Thus, the study suggested no meaningful difference between a single dose of PEG-F and multiple doses of FIL for shortening the duration of SN following chemotherapy.

### 3.4. Outcome of FIL versus Bio-F

**Figure 4:**
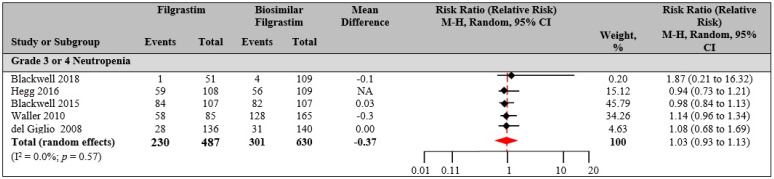
Filgrastim versus biosimilar filgrastim. Risk estimates: duration of severe neutropenia (1117 total patients; filgrastim, *n* = 487; pegfilgrastim, *n* = 630). Random effects meta-analysis was performed for the outcomes to compare data from clinical trials for filgrastim versus biosimilar filgrastim, and the relative risk was determined.

Five studies [76,77,78,79,80,81] were conducted for evaluating FIL versus Bio-F: a total of 1117 patients were enrolled in total, with FIL administered to 487 patients. The results of the primary endpoint outcome indicated that the pooled mean difference in DSN between FIL and Bio-F were small and not statistically significant (mean difference 0.37; RR = 1.029, 95% CI 0.933–1.134, I^2^ = 0.0%) (Figure 4). No clinically meaningful differences were observed regarding any other secondary efficacy parameter. The difference in mean ANC depth between FIL and Bio-F was 0.05 × 10^9^/L, and the time to ANC nadir for patients for both the treatments were same. The proportion of patients experiencing FN was similar between the FIL and Bio-F groups (RR = 0.87, 95% CI 0.56–1.35, I^2^ = 0.0%). Safety outcomes were also found to be similar between FIL and Bio-F, including bone pain (RR 1.18; 95% CI 0.68 to 2.05) and myalgia events (RR 1.05; 95% CI 0.675 to 1.631). The overall analysis therefore revealed no significant difference between FIL and Bio-F.

## 4. Discussion

In the present meta-analysis, the comparative effectiveness of G-CSF drugs (FIL versus PCT/NT; FIL versus PEG-F; FIL versus Bio-F) for cancer patients receiving chemotherapy in 56 studies containing 13,058 patients were evaluated, using FN, grade 3 or 4 neutropenia and bone pain as indicators. The 42 RCTs, 2 NRCTs and 12 observational studies in CIN, AML, SCN, BMT and PBCL indication identified in this study were between 1991 and 2019. The studies described here included multiple types of cancer, multiple geographic locations, different age groups and different healthcare systems, thereby increasing the generalizability of these results. The FIL dose, frequency and duration were provided in most studies, i.e., 52/56 studies (93%), and were varied across the studies. However, extensive study needs to be done to understand the effect of the modification of dose, frequency and duration of FIL.

Our meta-analyses confirm and strengthen the previous evidence that PP with FIL in U.S. approved indications is effective in reducing chemotherapy-associated FN. In particular for CIN, the indication of the risk of febrile neutropenia in filgrastim versus placebo/no treatment was not statistically significant (RR 0.58, 95% CI 0.50 to 0.67). However, the rate of reducing grade 3 or 4 neutropenia in filgrastim was statistically significant when compared to placebo/no treatment (RR 0.60, 95% CI 0.37 to 0.99). Regarding secondary outcomes, hospitalization was 10 days in FIL patients, compared to 14 days in PCT/NT patients, and documented infection associated with several malignancies and treatment regimens was less with FIL versus PCT/NT. These findings are consistent with the previous observations [31,85]. A study by Lyman et al. [86] suggested a non-significant increase in OS in patients receiving FIL versus PCT/NT, but in our analysis and in the study by Dale et al. [31], none of the studies reported a difference in the survival rates of FIL versus placebo. Additionally, the follow-up time was not consistently reported among the studies.

In AML, the median duration of neutropenia was observed to be shorter with FIL, compared to PCT/NT (10-12 days versus 15-17 days). FIL also shortened the time to ANC recovery in three RCTs [53,56,58] following induction chemotherapy and one NRCT [59] following consolidation chemotherapy, with no significant difference in the documented sources of infection. Similar results were reported by Bradley and colleagues [87] in their retrospective medical records review study and requires substantially larger trials. We did not find any OS benefit in patients receiving induction therapy in three RCTs [53,54,58].

In SCN indication, FIL increased the median ANC count and resulted in approximately 50% reduction in incidence and duration of infection-related events [60]. These results were confirmed by a long-term follow-up study of nearly 853 patients treated with daily or alternate day FIL injection [88].

When compared to PEG-F, our findings demonstrated that no notable differences were found between PEG-F and FIL in terms of efficacy and safety. Various studies have demonstrated that a single, fixed dose of PEG-F supports neutrophil recovery in a manner similar to a daily dose of FIL and is cost-effective [8,89]. However, a pooled analysis of trials suggests that PEG-F could be, in fact, advantageous in this respect. The safety profile of PEG-F was similar to FIL, and the mean frequency of bone pain among patients was less in the PEG-F arm than in the FIL arm.

The results from this meta-analysis showed that Bio-F is similar to reference FIL when assessed from the available RCTs. DSN was the primary endpoint when comparing FIL with Bio-F. The pooled mean difference in DSN between reference FIL and Bio-F was statistically not significant (0.37 d; 95% CI 0.933–1.134). For secondary efficacy endpoints, no clinically meaningful differences were observed. A meta-analysis including studies in patient with breast cancer who received either reference FIL/PEG-F or biosimilar FIL also reported a non-significant difference in DSN (0.06 d; 95% CI 0.05–0.17). The meta-analysis included eight RCTs, where FIL found reference in five studies and PEG-F in three studies [90]. The results from the study showed a difference of 0.09 (95% CI 0.15–0.05), indicating a very small difference between the two studies. The results we reported support the role of Bio-F in supportive care of patients receiving myelosuppressive chemotherapy. This presents an opportunity of cost savings associated with the use of biosimilars as reported in cost efficiency analysis studies [91,92,93,94]. These cost-savings can improve the financial sustainability of the healthcare system and increase patient’s uptake of biologics.

Bone pain is one of the common AEs associated with G-CSF drugs FIL and PEG-F [95] and is also an indicator of G-CSF drug tolerance. FIL showed a non-significant increase in the incidence of bone pain (RR 1.75, 95% CI 0.94 to 3.28) when compared to PCT/NT. Additionally, when compared with PEG-F, a non-significant increase in the incidence of bone pain was observed with FIL (RR 0.56, 95% CI 0.26 to 1.19). These results are consistent with results of other meta-analyses [85,96]. Another ADR observed was thrombocytopenia, which developed as an undesirable side-effect after PEG-F administration [69,74]. This is more likely due to bone marrow exhaustion from pre-treatment with highly myelosuppressive agents [97]. AEs, such as pulmonary toxicity in GCM tumor patients [37] and splenomegaly in SCN patients [60], were also observed.

The study shares a few limitations of most of the meta-analyses. First, there are differences in the baseline patient characteristics (e.g., cancer type, and patient history), differences in study design (dose and timing of intervention), changes in concomitant treatment strategies (prophylactic antibiotic use) and differences in the definition of FN and efficacy outcomes. Second, systematic reviews and meta-analysis rely on the quality of the included studies. Most included studies were considered at low risk of bias; patients were usually not blinded. Thirdly, studies of BMT and PBPC were excluded, due to sample size. Fourth, the number of studies included for FIL-Bio-F comparison was small; to limit the potential for bias, efforts were made to include all relevant studies in this meta-analysis, including searching reference list and online databases of registered clinical trials.

## 5. Conclusions

In conclusion, this is the largest dataset evaluating simultaneously the efficacy of FIL versus PCT/NT, FIL versus PEG-F, and FIL versus Bio-F in U.S. approved indications. The findings from this study demonstrated that PP with FIL, when compared to placebo, is effective in reducing the risk of FN in adults undergoing myelosuppressive chemotherapy. In addition, no notable differences were found between PEG-F and FIL. Bone pain was the most reported AE of FIL across all indications. Clinically no significant differences were observed in the efficacy and safety between reference FIL and biosimilar medicines. More head-to-head trials and real-world data analyses are suggested to validate the comparative findings.

## Figures and Tables

**Table 1:** Incidence of FN and Grade 3 or 4 neutropenia in CIN (RCT trials).

Study	Tumor Type	Filgrastim Intervention and Patient Number	FN Incidence	Grade 3 or 4 Neutropenia Incidence	Definition of FN
**FIL vs. PCT/NT**					
Crawford et al., 2005 [33]	SCLC	N = 231 Filgrastim = 111 Placebo = 120	Incidence of FN 38% vs. 74% *p* = 0.001	Grade 4 neutropenia incidence in cycle 1 82% vs. 97%	≥38.2 °C
Timmer-Bonte et al., 2005 [36]	SCLC	N = 175 Filgrastim = 90 Placebo = 85	Incidence of FN in cycle 1:10% vs. 24% Incidence of FN 2 to 5 cycles: 11% vs. 17% *p* = 0.001	NR	≥38.2 °C
Doorduijun et al., 2003 [34]	NHL	N = 389 Filgrastim = 197 Placebo = 192	Incidence of FN 36.5% vs. 44.8% *p* = 0.04	NR	≥38.2 °C
Obsy et al., 2003 [35]	NHL	N = 455 Filgrastim = 226 Placebo = 229	Granulocytopenic fever (<0.5 × 10^9^/L) CHOP arms 34% vs. 50% *p* = not reported	Granulocytopenia (<0.5 × 10^9^/L) CHOP arms 55% vs. 89%	>38.5 °C once or >38.0 °C twice in 4 h
Obsy et al., 2003 [35]	NHL	N = 455 Filgrastim = 226 Placebo = 229	Granulocytopenic fever (<0.5 × 10^9^/L) CNOP arms 32% vs. 50% *p* = not reported	Granulocytopenia (<0.5 × 10^9^/L) CNOP arms 64% vs. 86%	>38.5 °C once or >38.0 °C twice in 4 h
Papaldo et al., 2003 [39]	Breast Cancer	N = 503 Filgrastim = 254 No Filgrastim = 249	Incidence of FN 1.2% vs. 6.6% *p* = 0.004	Grade 3/4 neutropenia: 28.6 vs. 81.6% *p* < 0.00001	NR
Fossa et al., 1998 [37]	Germ Cell	N = 259 Filgrastim = 129 No Filgrastim = 130	Incidence of FN 20% vs. 30% *p* < 0.052	NR	NR
Geissler et al., 1997 [40]	ALL	N = 51 Filgrastim = 25 Placebo = 26	Incidence of FN 12% vs. 42% *p* = not reported	NR	>38.0 °C
Ottmann et al., 1995 [52]	ALL	N = 76 Filgrastim = 37 No filgrastim = 39	Duration of prolonged neutropenia incidence 22% vs. 42% *p* = not reported	NR	>38.0 °C
Zinzani et al., 1997 [42]	NHL	N = 149 Filgrastim = 77 No filgrastim = 72	NR	Grade 4 neutropenia incidence 23.0% vs. 55.5% *p* = 0.00005	NR
Gebbia et al., 1993 [51]	Breast cancer	N = 86 Filgrastim = 43 Placebo = 43	Incidence of FN 12% vs. 32% *p* < 0.05	Grade 3 and 4 neutropenia 18% vs. 42% *p* < 0.05	>38.0 °C
Trillet-Lenoir et al., 1993 [45]	SCLC	N = 129 Filgrastim = 65 Placebo = 64	Incidence of FN 26% vs. 53% *p* = 0.002	NR	≥38.2 °C
Crawford et al., 1991 [7]	SCLC	N = 199 Filgrastim = 95 Placebo = 104	Incidence of FN in cycle 1 28% vs. 57% *p* < 0.001 FN incidence across 6 cycles 40% vs. 77% *p* < 0.001	Grade 4 neutropenia incidence in cycle 1 84% vs. 98% *p* = 0.001	≥38.2 °C
**FIL vs. PEG-F**					
Park et al., 2017 [66]	Breast Cancer	N = 74 Filgrastim = 38 DA 3031 = 36	Incidence of FN 7.9% vs. 17.1%	NR	NR
Kubo et al., 2016 [70]	NHL	N = 111 Filgrastim = 56 Pegfilgrastim = 55	Incidence of FN 55.6% vs. 56.6% *p* = not reported	NR	<37.5 °C
Zhang et al., 2015 [68]	Breast Cancer	N = 171 Filgrastim = 43 PEG 60 μg/kg = 43 PEG 100 μg/kg = 43 PEG 120 μg/kg = 42	Incidence of FN 11.63% vs. 6.98% vs. 4.65% vs. 11.90%	NR	≥38.2 °C
Park et al., 2013 [67]	Breast Cancer	N = 61 Filgrastim = 21 DA-3031 3.6 mg = 20 DA-3031 6 mg = 20	Incidence of FN9.5% vs. 15% vs. 5%	NR	NR
Green et al., 2003 [8]	Breast Cancer	N = 152 Filgrastim: 75 Pegfilgrastim: 77	Incidence of FN 15% vs. 9%	NR	≥38.2 °C
Grigg et al., 2003 [73]	NHL	N = 50 Filgrastim: 22 Pegfilgrastim: 27	Incidence of FN in cycle 1 was 0% vs. 15%	NR	≥38.2 °C
Vose et al., 2003 [74]	NHL	N = 60 Filgrastim: 31 Pegfilgrastim: 29	Incidence of FN 19% vs. 21%	Incidence of grade 4 neutropenia 68% vs. 69%	≥38.2 °C
Holmes et al., 2002 [9]	Breast Cancer	N = 296 Filgrastim: 147 Pegfilgrastim: 149	Incidence of FN 18% vs. 9%	Incidence of grade 4 neutropenia in cycle 1 79% vs. 77%	≥38.2 °C
Holmes et al., 2002 [19]	Breast Cancer	N = 125 Filgrastim = 25 PEG 30 μg/kg = 19 PEG 60 μg/kg = 60 PEG 100 μg/kg = 46	Incidence of FN 4% vs. 21% vs. 8% vs. 7%	Incidence of grade 4 neutropenia 76% vs. 95% vs. 90% vs. 74%	≥38.2 °C
**FIL vs. Bio-F**					
Blackwell et al., 2018 [76]	Breast Cancer	N = 213 Filgrastim: 51 Switched biosimilar: 109 EP2006: 53	Incidence of FN across cycles 2–6 0% vs. 3.4%	NR	≥38.3 °C
Hegg et al., 2016 [80]	Breast Cancer	N = 217 Filgrastim: 108 Biosimilar filgrastim: 109	NR	NR	≥38.2 °C
Blackwell et al., 2015 [77]	Breast Cancer	N = 214 Filgrastim: 107 EP 2006: 107	NR	NR	≥38.3 °C
Waller et al., 2010 [81]	Breast Cancer	N = 278 Filgrastim: 95 Biosimilar filgrastim: 183	Incidence of FN in cycle 1–3 2.4% vs. 2.4%	NR	≥38.5 °C
del Giglio et al., 2008 [78]	Breast Cancer	N = 348 Filgrastim: *n* = 136 XM02: *n* = 140 Placebo/XM02: *n* = 72	FN incidence 20.7% vs. 22.1% vs. 41.7%	NR	>38.5 °C

Abbreviations: FIL: filgrastim; FN: febrile neutropenia; PCT/NT: placebo/no treatment; NR: not reported; SCLC: small-cell lung carcinoma; NHL: non-Hodgkin lymphoma; ALL: acute lymphoblastic leukemia.

## Data Availability

The data generated from the study are clearly presented and discussed in the manuscript.

## Supplementary Materials

The following are available online at https://www.mdpi.com/article/10.3390/biology10101069/s1, Table S1: Full electronic search in MEDLINE database through 31 July 2020; Table S2: Key characteristics of included studies of filgrastim versus placebo/no treatment; Table S3: Key characteristics of included studies of filgrastim versus pegfilgrastim; Table S4: Key characteristics of included studies of filgrastim versus biosimilar filgrastim; Table S5: Baseline and clinical patients’ characteristics: (filgrastim vs. placebo controlled trial/no treatment); Table S6: Baseline and clinical patients’ characteristics (filgrastim vs. pegfilgrastim); Table S7: Baseline and clinical patients’ characteristics (filgrastim vs. biosimilar filgrastim); Table S8: Type of trial reported (filgrastim vs. placebo controlled trial/no treatment; Table S9: Type of trial reported (filgrastim vs. pegfilgrastim); Table S10: Type of trial reported (filgrastim vs. biosimilar filgrastim); Table S11: Key characteristics of studies that compared filgrastim with placebo or no treatment by indication and study type; Table S12: Key characteristics of studies that compared filgrastim with pegfilgrastim by indication and study type; Table S13: Key characteristics of studies that compared filgrastim with biosimilar filgrastim by indication and study type; Figure S1: Cochrane Collaboration Risk of Bias Assessment Tool version 2 (RoB 2); Figure S2: Funnel plots for filgrastim versus placebo/no treatment; Figure S3: Funnel plots for filgrastim versus pegfilgrastim.

## References

[1] Kuderer N.M., Dale D.C., Crawford J., Cosler L.E., Lyman G.H. (2006). Mortality, morbidity, and cost associated with febrile neutropenia in adult cancer patients. Cancer.

[2] Rossi L., Tomao F., Russo G.L., Papa A., Zoratto F., Marzano R., Basso E., Giordani E., Verrico M., Ricci F. (2013). Efficacy and safety analysis of once per cycle pegfilgrastim and daily lenograstim in patients with breast cancer receiving adjuvant myelosuppressive chemotherapy FEC 100: A pilot study. Ther. Clin. Risk Manag..

[3] Lyman G.H., Morrison V.A., Dale D.C., Crawford J., Delgado D.J., Fridman M. (2003). Risk of febrile neutropenia among patients with intermediate-grade non-Hodgkin’s lymphoma receiving CHOP chemotherapy. Leuk. Lymphoma.

[4] Wildiers H., Reiser M. (2011). Relative dose intensity of chemotherapy and its impact on outcomes in patients with early breast cancer or aggressive lymphoma. Crit. Rev. Oncol..

[5] Chatta G.S., Price T.H., Allen R.C., Dale D.C. (1994). Effects of In Vivo recombinant methionyl human granulocyte colony-stimulating factor on the neutrophil response and peripheral blood colony-forming cells in healthy young and elderly adult volunteers. Blood.

[6] Groopman J.E., Molina J.-M., Scadden D.T. (1989). Hematopoietic growth factors. N. Engl. J. Med..

[7] Crawford J., Ozer H., Stoller R., Johnson D., Lyman G., Tabbara I., Kris M., Grous J., Picozzi V., Rausch G. (1991). Reduction by granulocyte colony-stimulating factor of fever and neutropenia induced by chemotherapy in patients with small-cell lung cancer. N. Engl. J. Med..

[8] Green M., Koelbl H., Baselga J., Galid A., Guillem V., Gascon P., Siena S., Lalisang R., Samonigg H., Clemens M. (2003). A randomized double-blind multicenter phase III study of fixed-dose single-administration pegfilgrastim versus daily filgrastim in patients receiving myelosuppressive chemotherapy. Ann. Oncol..

[9] Holmes F., O’shaughnessy J., Vukelja S., Jones S., Shogan J., Savin M., Glaspy J., Moore M., Meza L., Wiznitzer I. (2002). Blinded, randomized, multicenter study to evaluate single administration pegfilgrastim once per cycle versus daily filgrastim as an adjunct to chemotherapy in patients with high-risk stage II or stage III/IV breast cancer. J. Clin. Oncol..

[10] Crawford J., Althaus B., Armitage J., Balducci L., Bennett C., Blayney D.W., Cataland S.R., Dale D.C., Demetri G.D., Erba H.P. (2007). Myeloid growth factors: Clinical practice guidelines in oncology™. J. Natl. Compr. Cancer Netw..

[11] Carmona-Bayonas A., Jimenez-Fonseca P., De Castro E.M., Mata E., Biosca M., Custodio A., Espinosa J., Vázquez E.G., Henao F., De La Peña F.A. (2018). SEOM clinical practice guideline: Management and prevention of febrile neutropenia in adults with solid tumors (2018). Clin. Transl. Oncol..

[12] Taplitz R.A., Kennedy E.B., Bow E.J., Crews J., Gleason C., Hawley D.K., Langston A.A., Nastoupil L.J., Rajotte M., Rolston K. (2018). Outpatient management of fever and neutropenia in adults treated for malignancy: American society of clinical oncology and infectious diseases society of America clinical practice guideline update. J. Clin. Oncol..

[13] Klastersky J., de Naurois J., Rolston K., Rapoport B., Maschmeyer G., Aapro M., Herrstedt J. (2016). Management of febrile neutropaenia: ESMO clinical practice guidelines. Ann. Oncol..

[14] Baden L.R., Swaminathan S., Angarone M., Blouin G., Camins B.C., Casper C., Cooper B., Dubberke E.R., Engemann A.M., Freifeld A.G. (2016). Prevention and treatment of cancer-related infections, version 2.2016, NCCN clinical practice guidelines in oncology. J. Natl. Compr. Cancer Netw..

[15] Clark O.A., Lyman G.H., Castro A.A., Clark L.G., Djulbegovic B. (2005). Colony-stimulating factors for chemotherapy-induced febrile neutropenia: A meta-analysis of randomized controlled trials. J. Clin. Oncol..

[16] Welte K., Gabrilove J., Bronchud M.H., Platzer E., Morstyn G. (1996). Filgrastim (r-metHuG-CSF): The first 10 years. Blood.

[17] Amgen Inc (2000). Neupogen (Filgrastim) Prescribing Information.

[18] Yang B.-B., Savin M.A., Green M. (2012). Prevention of chemotherapy-induced neutropenia with pegfilgrastim: Pharmacokinetics and patient outcomes. Chemotherapy.

[19] Holmes F., Jones S., O’shaughnessy J., Vukelja S., George T., Savin M., Richards D., Glaspy J., Meza L., Cohen G. (2002). Comparable efficacy and safety profiles of once-per-cycle pegfilgrastim and daily injection filgrastim in chemotherapy-induced neutropenia: A multicenter dose-finding study in women with breast cancer. Ann. Oncol..

[20] Pinto L., Liu Z., Doan Q., Bernal M., Dubois R., Lyman G. (2007). Comparison of pegfilgrastim with filgrastim on febrile neutropenia, grade IV neutropenia and bone pain: A meta-analysis of randomized controlled trials. Curr. Med Res. Opin..

[21] Rastogi S., Shukla S., Sharma A.K., Sarwat M., Srivastava P., Katiyar T., Kalaiselvan V., Singh G.N. (2020). Towards a comprehensive safety understanding of granulocyte-colony stimulating factor biosimilars in treating chemotherapy associated febrile neutropenia: Trends from decades of data. Toxicol. Appl. Pharmacol..

[22] He K., Chen H., Gwise T., Casak S., Lemery S., Keegan P., Pazdur R., Sridhara R. (2016). Statistical considerations in evaluating a biosimilar product in an oncology clinical study. Clin. Cancer Res..

[23] Daller J. (2016). Biosimilars: A consideration of the regulations in the United States and European union. Regul. Toxicol. Pharmacol..

[24] Kuderer N.M., Dale D.C., Crawford J., Lyman G.H. (2007). Impact of primary prophylaxis with granulocyte colony-stimulating factor on febrile neutropenia and mortality in adult cancer patients receiving chemotherapy: A systematic review. J. Clin. Oncol..

[25] Lyman G.H., Dale D.C., Culakova E., Poniewierski M.S., Wolff D.A., Kuderer N.M., Huang M., Crawford J. (2013). The impact of the granulocyte colony-stimulating factor on chemotherapy dose intensity and cancer survival: A systematic review and meta-analysis of randomized controlled trials. Ann. Oncol..

[26] Almenar Cubells D., Bosch Roig C., Jimenez Orozco E., Alvarez R., Cuervo J., Diaz Fernandez N., Sanchez Heras A., Galan Brotons A., Giner Marco V., Codes M. (2013). Effectiveness of daily versus non-daily granulocyte colony-stimulating factors in patients with solid tumours undergoing chemotherapy: A multivariate analysis of data from current practice. Eur. J. Cancer Care.

[27] Morrison V.A., Wong M., Hershman D., Campos L.T., Ding B., Malin J. (2007). Observational study of the prevalence of febrile neutropenia in patients who received filgrastim or pegfilgrastim associated with 3–4 week chemotherapy regimens in community oncology practices. J. Manag. Care Pharm..

[28] Naeim A., Henk H.J., Becker L., Chia V., Badre S., Deeter R.G. (2010). Pegfilgrastim use associated with lower risk of hospitalization than filgrastim use: A retrospective US claims analysis. Blood.

[29] Naeim A., Henk H.J., Becker L., Chia V., Badre S., Li X., Deeter R. (2013). Pegfilgrastim prophylaxis is associated with a lower risk of hospitalization of cancer patients than filgrastim prophylaxis: A retrospective United States claims analysis of granulocyte colony-stimulating factors (G-CSF). BMC Cancer.

[30] Brito M., Esteves S., André R., Isidoro M., Moreira A. (2016). Comparison of effectiveness of biosimilar filgrastim (Nivestim™), reference Amgen filgrastim and pegfilgrastim in febrile neutropenia primary prevention in breast cancer patients treated with neo (adjuvant) TAC: A non-interventional cohort study. Support. Care Cancer.

[31] Dale D.C., Crawford J., Klippel Z., Reiner M., Osslund T., Fan E., Morrow P.K., Allcott K., Lyman G.H. (2018). A systematic literature review of the efficacy, effectiveness, and safety of filgrastim. Support. Care Cancer.

[32] Sterne J.A.C., Savović J., Page M.J., Elbers R.G., Blencowe N.S., Boutron I., Cates C.J., Cheng H.-Y., Corbett M.S., Eldridge S.M. (2019). RoB 2: A revised tool for assessing risk of bias in randomised trials. BMJ.

[33] Crawford J., Glaspy J.A., Stoller R.G., Tomita D.K., Vincent M.E., McGuire B.W., Ozer H. (2005). Final results of a placebo-controlled study of filgrastim in small-cell lung cancer: Exploration of risk factors for febrile neutropenia. Support. Cancer Ther..

[34] Doorduijn J., Van Der Holt B., Van Imhoff G., Van Der Hem K., Kramer M., Van Oers M., Ossenkoppele G., Schaafsma M., Verdonck L., Verhoef G. (2003). CHOP compared with CHOP plus granulocyte colony-stimulating factor in elderly patients with aggressive non-hodgkin’s lymphoma. J. Clin. Oncol..

[35] Osby E., Hagberg H., Kvaløy S., Teerenhovi L., Anderson H., Cavallin-Ståhl E., Holte H., Myhre J., Pertovaara H., Björkholm M. (2003). CHOP is superior to CNOP in elderly patients with aggressive lymphoma while outcome is unaffected by filgrastim treatment: Results of a Nordic lymphoma group randomized trial. Blood J. Am. Soc. Hematol..

[36] Timmer-Bonte J.N., De Boo T.M., Smit H.J., Biesma B., Wilschut F.A., Cheragwandi S.A., Termeer A., Hensing C.A., Akkermans J., Adang E.M. (2005). Prevention of chemotherapy-induced febrile neutropenia by prophylactic antibiotics plus or minus granulocyte colony-stimulating factor in small-cell lung cancer: A Dutch randomized phase III study. J. Clin. Oncol..

[37] Fosså S.D., Kaye S.B., Mead G.M., Cullen M., De Wit R., Bodrogi I., Van Groeningen C.J., De Mulder P.H., Stenning S., Lallemand E. (1998). Filgrastim during combination chemotherapy of patients with poor-prognosis metastatic germ cell malignancy. J. Clin. Oncol..

[38] Larson R.A., Dodge R.K., Linker C.A., Stone R.M., Powell B.L., Lee E.J., Schulman P., Davey F.R., Frankel S.R., Bloomfield C.D. (1998). A randomized controlled trial of filgrastim during remission induction and consolidation chemotherapy for adults with acute lymphoblastic leukemia: CALGB study 9111. Blood.

[39] Papaldo P., Lopez M., Cortesi E., Cammilluzzi E., Antimi M., Terzoli E., Lepidini G., Vici P., Barone C., Ferretti G. (2003). Addition of either lonidamine or granulocyte colony-stimulating factor does not improve survival in early breast cancer patients treated with high-dose epirubicin and cyclophosphamide. J. Clin. Oncol..

[40] Geissler K., Koller E., Hubmann E., Niederwieser D., Hinterberger W., Geissler D., Kyrle P., Knöbl P., Pabinger I., Thalhammer R. (1997). Granulocyte colony-stimulating factor as an adjunct to induction chemotherapy for adult acute lymphoblastic leukemia—a randomized phase-III study. Blood J. Am. Soc. Hematol..

[41] Michon J., Hartmann O., Bouffet E., Meresse V., Coze C., Rubie H., Bordigoni P., Cattiaux E., Ward N., Bernard J.-L. (1998). An open-label, multicentre, randomised phase 2 study of recombinant human granulocyte colony-stimulating factor (filgrastim) as an adjunct to combination chemotherapy in paediatric patients with metastatic neuroblastoma. Eur. J. Cancer.

[42] Zinzani P.L., Pavone E., Storti S., Moretti L., Fattori P.P., Guardigni L., Falini B., Gobbi M., Gentilini P., Lauta V.M. (1997). Randomized trial with or without granulocyte colony-stimulating factor as adjunct to induction VNCOP-B treatment of elderly high-grade non-Hodgkin’s lymphoma. Blood J. Am. Soc. Hematol..

[43] Pui C.-H., Boyett J.M., Hughes W.T., Rivera G.K., Hancock M.L., Sandlund J.T., Synold T., Relling M.V., Ribeiro R.C., Crist W.M. (1997). Human granulocyte colony-stimulating factor after induction chemotherapy in children with acute lymphoblastic leukemia. N. Engl. J. Med..

[44] Maher D.W., Lieschke G.J., Green M., Bishop J., Stuart-Harris R., Wolf M., Sheridan W.P., Kefford R.F., Cebon J., Olver I. (1994). Filgrastim in patients with chemotherapy-induced febrile neutropenia: A double-blind, placebo-controlled trial. Ann. Intern. Med..

[45] Trillet-Lenoir V., Green J., Manegold C., Von Pawel J., Gatzemeier U., Lebeau B., Depierre A., Johnson P., Decoster G., Tomita D. (1993). Recombinant granulocyte colony stimulating factor reduces the infectious complications of cytotoxic chemotherapy. Eur. J. Cancer.

[46] Blayney D.W., McGuire B.W., Cruickshank S.E., Johnson D.H. (2005). Increasing chemotherapy dose density and intensity: Phase I trials in non-small cell lung cancer and non-Hodgkin’s lymphoma. Oncologist.

[47] Altwairgi A., Hopman W., Mates M. (2013). Real-world impact of granulocyte-colony stimulating factor on febrile neutropenia. Curr. Oncol..

[48] Chen J., Pan Y. (2017). The safety and clinical efficacy of recombinant human granulocyte colony stimulating factor injection for colon cancer patients undergoing chemotherapy. Rev. Assoc. Méd. Bras..

[49] Hershman D., Hurley D., Wong M., Morrison V.A., Malin J.L. (2009). Impact of primary prophylaxis on febrile neutropenia within community practices in the US. J. Med. Econ..

[50] Gilad J., Riesenberg K., Mermershtain W., Borer A., Porath A., Schlaeffer F. (1999). Granulocyte-colony stimulating factor for the prevention of chemotherapy-induced febrile neutropenia in the adult cancer patient population of Southern Israel. Support. Care Cancer.

[51] Gebbia V., Testa A., Valenza R., Borsellino N., Cipolla C., Cannata G., Curto G., Latteri M., Florena M., Gebbia N. (1993). A prospective evaluation of the activity of human granulocyte-colony stimulating factor on the prevention of chemotherapy-related neutropenia in patients with advanced carcinoma. J. Chemother..

[52] Ottmann O., Hoelzer D., Gracien E., Ganser A., Kelly K., Reutzel R., Lipp T., Busch F., Schwonzen M., Heil G. (1995). Concomitant granulocyte colony-stimulating factor and induction chemoradiotherapy in adult acute lymphoblastic leukemia: A randomized phase III trial. Blood.

[53] Usuki K., Urabe A., Masaoka T., Ohno R., Mizoguchi H., Hamajima N., Miyazaki T., Niitsu Y., Yoshida Y., Miura A. (2002). Efficacy of granulocyte colony-stimulating factor in the treatment of acute myelogenous leukaemia: A multicentre randomized study. Br. J. Haematol..

[54] Beksac M., Ali R., Ozcelik T., Özcan M., Ozcebe O., Bayık M., Paydas S., Büyükasik Y., Ilhan O., Ozkalemkas F. (2011). Short and long term effects of granulocyte colony-stimulating factor during induction therapy in acute myeloid leukemia patients younger than 65: Results of a randomized multicenter phase III trial. Leuk. Res..

[55] Heil G., Hoelzer D., Sanz M.A., Lechner K., Noens L., Szer J., Ganser A., Matcham J., Renwick J. (2006). Long-term survival data from a phase 3 study of filgrastim as an adjunct to chemotherapy in adults with de novo acute myeloid leukemia. Leukemia.

[56] Godwin J.E., Kopecky K.J., Head D.R., Willman C.L., Leith C.P., Hynes H.E., Balcerzak S.P., Appelbaum F.R. (1998). A double-blind placebo-controlled trial of granulocyte colony-stimulating factor in elderly patients with previously untreated acute myeloid leukemia: A Southwest oncology group study (9031). Blood J. Am. Soc. Hematol..

[57] Harousseau J., Witz B., Lioure B., Hunault-Berger M., Desablens B., Delain M., Guilhot F., Le Prise P., Abgrall J., Deconinck E. (2000). Granulocyte colony-stimulating factor after intensive consolidation chemotherapy in acute myeloid leukemia: Results of a randomized trial of the groupe ouest-est leucémies aigues myeloblastiques. J. Clin. Oncol..

[58] Heil G., Hoelzer D., Sanz M.A., Lechner K., Liu Yin J.A., Papa G., Noens L., Szer J., Ganser A., O’Brien C. (1997). A randomized, double-blind, placebo-controlled, phase III study of filgrastim in remission induction and consolidation therapy for adults with de novo acute myeloid leukemia. Blood J. Am. Soc. Hematol..

[59] Moore J.O., Dodge R.K., Amrein P.C., Kolitz J., Lee E.J., Powell B., Godfrey S., Robert F., Schiffer C.A. (1997). Granulocyte colony-stimulating factor (filgrastim) accelerates granulocyte recovery after intensive postremission chemotherapy for acute myeloid leukemia with aziridinyl benzoquinone and mitoxantrone: Cancer and leukemia group B study 9022. Blood.

[60] Dale D.C., Bonilla M.A., Davis M.W., Nakanishi A.M., Hammond W.P., Kurtzberg J., Wang W., Jakubowski A., Winton E., Lalezari P. (1993). A randomized controlled phase III trial of recombinant human granulocyte colony-stimulating factor (filgrastim) for treatment of severe chronic neutropenia. Blood.

[61] Yilmaz D., Ritchey A.K. (2007). Severe neutropenia in children: A single institutional experience. J. Pediatric Hematol. Oncol..

[62] González-Vicent M., Madero L., Sevilla J., Ramirez M., Diaz M., Gonz L.M.M. (2004). A prospective randomized study of clinical and economic consequences of using G-CSF following autologous peripheral blood progenitor cell (PBPC) transplantation in children. Bone Marrow Transplant..

[63] Stahel R.A., Jost L.M., Honegger H., Betts E., Goebel M.E., Nagler A. (1997). Randomized trial showing equivalent efficacy of filgrastim 5 μg/kg/d and 10 μg/kg/d following high-dose chemotherapy and autologous bone marrow transplantation in high-risk lymphomas. J. Clin. Oncol..

[64] Damiani D., Fanin R., Silvestri F., Grimaz S., Infanti L., Geromin A., Cerno M., Michieli M., Rinaldi C., Savignano C. (1997). Randomized trial of autologous filgrastim-primed bone marrow transplantation versus filgrastim-mobilized peripheral blood stem cell transplantation in lymphoma patients. Blood J. Am. Soc. Hematol..

[65] Gertz M.A., Gastineau D.A., Lacy M.Q., Dispenzieri A., Hayman S.R., Kumar S.K., Dingli D., Leung N., Wolf R.C., Hogan W.J. (2010). SCT without growth factor in multiple myeloma: Engraftment kinetics, bacteremia and hospitalization. Bone Marrow Transplant..

[66] Park K.H., Park J.H., Kang S.Y., Kim H.Y., Park I.H., Park Y.H., Im Y.H., Lee H.J., Park S., Lee S. (2016). A randomized, multi-center, open-label, phase III study of once-per-cycle DA-3031, a pegylated G-CSF, in comparison with daily filgrastim in patients receiving TAC chemotherapy for breast cancer. Support. Care Cancer.

[67] Park K.H., Sohn J., Lee S., Kang S.Y., Kim H.Y., Im Y.H., Lee H.J., Hong D.S., Park S., Shin S.H. (2013). A randomized, multi-center, open-label, phase II study of once-per-cycle DA-3031, a biosimilar pegylated G-CSF, compared with daily filgrastim in patients receiving TAC chemotherapy for early-stage breast cancer. Investig. New Drugs.

[68] Zhang W., Jiang Z., Wang L., Li C., Xia J. (2015). An open-label, randomized, multicenter dose-finding study of once-per-cycle pegfilgrastim versus daily filgrastim in Chinese breast cancer patients receiving TAC chemotherapy. Med. Oncol..

[69] Xu F., Zhang Y., Miao Z., Zeng X., Wu B., Cai L., Liu J., Wang S., Hu X., Zheng W. (2019). Efficacy and safety of mecapegfilgrastim for prophylaxis of chemotherapy-induced neutropenia in patients with breast cancer: A randomized, multicenter, active-controlled phase III trial. Ann. Transl. Med..

[70] Kubo K., Miyazaki Y., Murayama T., Shimazaki R., Usui N., Urabe A., Hotta T., Tamura K. (2016). A randomized, double-blind trial of pegfilgrastim versus filgrastim for the management of neutropenia during CHASE (R) chemotherapy for malignant lymphoma. Br. J. Haematol..

[71] Filon O., Nechaeva M., Burdaeva O., Vladimirov V.I., Lifirenko I., Kovalenko N.V., Kopp M.V., Matrosova M., Mukhametsina G., Panchenko S. (2015). Efficacy and safety of empegfilgrastim, a novel pegylated G-CSF: Results of complete analysis after 4 cycles of myelosuppressive chemotherapy in phase III double-dummy randomized clinical study. Am. Soc. Clin. Oncol..

[72] Salafet O.V., Chernovskaya T.V., Sheveleva L.P., Khorinko A.V., Prokopenko T.I., Nechaeva M.P., Burdaeva O.N., Matrosova M.P., Kovalenko N.V., Ovchinnikova E.G. (2013). Efficacy and safety of BCD-017, a novel pegylated filgrastim: Results of open-label controlled phase II study in patients with breast cancer receiving myelosuppressive chemotherapy. J. Clin. Oncol..

[73] Grigg A., Solal-Celigny P., Hoskin P., Taylor K. (2003). Open-label, randomized study of pegfilgrastim vs. daily filgrastim as an adjunct to chemotherapy in elderly patients with non-Hodgkin’s lymphoma. Leuk. Lymphoma.

[74] Vose J., Crump M., Lazarus H., Emmanouilides C., Schenkein D., Moore J., Frankel S., Flinn I., Lovelace W., Hackett J. (2003). Randomized, multicenter, open-label study of pegfilgrastim compared with daily filgrastim after chemotherapy for lymphoma. J. Clin. Oncol..

[75] Sierra J., Szer J., Kassis J., Herrmann R., Lazzarino M., Thomas X., Noga S.J., Baker N., Dansey R., Bosi A. (2008). A single dose of pegfilgrastim compared with daily filgrastim for supporting neutrophil recovery in patients treated for low-to-intermediate risk acute myeloid leukemia: Results from a randomized, double-blind, phase 2 trial. BMC Cancer.

[76] Blackwell K., Gascon P., Krendyukov A., Gattu S., Li Y., Harbeck N. (2018). Safety and efficacy of alternating treatment with EP2006, a filgrastim biosimilar, and reference filgrastim: A phase III, randomised, double-blind clinical study in the prevention of severe neutropenia in patients with breast cancer receiving myelosuppressive chemotherapy. Ann. Oncol..

[77] Blackwell K., Semiglazov V., Krasnozhon D., Davidenko I., Nelyubina L., Nakov R., Stiegler G., Singh P., Schwebig A., Kramer S. (2015). Comparison of EP2006, a filgrastim biosimilar, to the reference: A phase III, randomized, double-blind clinical study in the prevention of severe neutropenia in patients with breast cancer receiving myelosuppressive chemotherapy. Ann. Oncol..

[78] Del Giglio A., Eniu A., Ganea-Motan D., Topuzov E., Lubenau H. (2008). XM02 is superior to placebo and equivalent to Neupogen™ in reducing the duration of severe neutropenia and the incidence of febrile neutropenia in cycle 1 in breast cancer patients receiving docetaxel/doxorubicin chemotherapy. BMC Cancer.

[79] Engert A., Griskevicius L., Zyuzgin Y., Lubenau H., Del Giglio A. (2009). XM02, the first granulocyte colony-stimulating factor biosimilar, is safe and effective in reducing the duration of severe neutropenia and incidence of febrile neutropenia in patients with non-Hodgkin lymphoma receiving chemotherapy. Leuk. Lymphoma.

[80] Hegg R., Mattar A., Matos-Neto J.N.D., Pedrini J.L., Aleixo S.B., Rocha R.O., Cramer-Junior R.P., van-Eyll-Rocha S. (2016). A phase III, randomized, non-inferiority study comparing the efficacy and safety of biosimilar filgrastim versus originator filgrastim for chemotherapy-induced neutropenia in breast cancer patients. Clinics.

[81] Waller C.F., Semiglazov V.F., Tjulandin S., Bentsion D., Chan S., Challand R. (2010). A Phase III randomized equivalence study of biosimilar filgrastim versus amgen filgrastim in patients receiving myelosuppressive chemotherapy for breast cancer. Oncol. Res. Treat..

[82] Sivgin S., Karakus E., Keklik M., Zararsiz G., Solmaz M., Kaynar L., Eser B., Cetin M., Unal A. (2016). Evaluation of the efficacy and safety of original filgrastim (Neupogen^®^), biosimilar filgrastim (Leucostim^®^) and Lenograstim (Granocyte^®^) in CD34^+^ peripheral hematopoietic stem cell mobilization procedures for allogeneic hematopoietic stem cell transplant donors. Transfus. Apher. Sci..

[83] Skopec B., Skerget M., Zontar D., Zadnik V., Zver S. (2017). Filgrastim-alone versus pegylated filgrastim-alone for autologous peripheral blood stem cells mobilization in newly diagnosed multiple myeloma patients. Wien. Klin. Wochenschr..

[84] Yoshimura H., Hotta M., Nakanishi T., Fujita S., Nakaya A., Satake A., Ito T., Ishii K., Nomura S. (2017). Evaluation of a biosimilar granulocyte colony-stimulating factor (filgrastim XM02) for peripheral blood stem cell mobilization and transplantation: A single center experience in Japan. J. Blood Med..

[85] Wang Y., Chen L., Liu F., Zhao N., Xu L., Fu B., Li Y. (2019). Efficacy and tolerability of granulocyte colony-stimulating factors in cancer patients after chemotherapy: A systematic review and Bayesian network meta-analysis. Sci. Rep..

[86] Lyman G.H., Reiner M., Morrow P.K., Crawford J. (2015). The effect of filgrastim or pegfilgrastim on survival outcomes of patients with cancer receiving myelosuppressive chemotherapy. Ann. Oncol..

[87] Bradley A.M., Deal A.M., Buie L.W., Van Deventer H. (2012). Neutropenia-associated outcomes in adults with acute myeloid leukemia receiving cytarabine consolidation chemotherapy with or without granulocyte colony-stimulating factor. Pharmacother. J. Hum. Pharmacol. Drug Ther..

[88] Dale D.C., Cottle T.E., Fier C.J., Bolyard A.A., Bonilla M.A., Boxer L.A., Cham B., Freedman M.H., Kannourakis G., Kinsey S.E. (2003). Severe chronic neutropenia: Treatment and follow-up of patients in the severe chronic neutropenia international registry. Am. J. Hematol..

[89] Sehouli J., Goertz A., Steinle T., Dubois R., Plesnila-Frank C., Lalla A., von Minckwitz G. (2010). Pegfilgrastim vs. filgrastim in primary prophylaxis of febrile neutropenia in patients with breast cancer after chemotherapy: A cost-effectiveness analysis for Germany. Dtsch. Med. Wochenschr..

[90] Botteri E., Krendyukov A., Curigliano G. (2018). Comparing granulocyte colony-stimulating factor filgrastim and pegfilgrastim to its biosimilars in terms of efficacy and safety: A meta-analysis of randomised clinical trials in breast cancer patients. Eur. J. Cancer.

[91] Aitken M. (2016). Delivering on the Potential of Biosimilar Medicines. The Role of Functioning Competitive Markets.

[92] Aapro M., Cornes P., Abraham I. (2012). Comparative cost-efficiency across the european G5 countries of various regimens of filgrastim, biosimilar filgrastim, and pegfilgrastim to reduce the incidence of chemotherapy-induced febrile neutropenia. J. Oncol. Pharm. Pract..

[93] Sun D., Andayani T.M., Altyar A., MacDonald K., Abraham I. (2015). Potential cost savings from chemotherapy-induced febrile neutropenia with biosimilar filgrastim and expanded access to targeted antineoplastic treatment across the european union G5 countries: A simulation study. Clin. Ther..

[94] Tabernero J., Vyas M., Giuliani R., Arnold D., Cardoso F., Casali P.G., Cervantes A., Eggermont A.M., Eniu A., Jassem J. (2016). Biosimilars: A position paper of the European Society for Medical Oncology, with particular reference to oncology prescribers. ESMO Open.

[95] Abboud C.N., Lang N., Fung H., Lammerich A., Buchner A., Liu P., Mueller U., Pettengell R., Diel I.J., Link H. (2018). Real-world safety experience of tevagrastim/ratiograstim/biograstim and tbo-filgrastim, short-acting recombinant human granulocyte colony-stimulating factors. Support. Care Cancer.

[96] Yang J., Yu S., Yang Z., Yan Y., Chen Y., Zeng H., Ma F., Shi Y., Shi Y., Zhang Z. (2019). Efficacy and safety of supportive care biosimilars among cancer patients: A systematic review and meta-analysis. BioDrugs.

[97] Te Poele E.M., Kamps W.A., Tamminga R.Y., Leeuw J.A., Postma A., de Bont E.S. (2005). Pegfilgrastim in pediatric cancer patients. J. Pediatric Hematol. Oncol..

