# Supplementary material for: Efficacy and Safety of Filgrastim and Its Biosimilars to Prevent Febrile Neutropenia in Cancer Patients: A Prospective Study and Meta-Analysis
Source: Biology (Basel). 2021 Oct 19;10(10):1069. doi: 10.3390/biology10101069 (PMC8533340; doi:10.3390/biology10101069)
Supplement: Supplementary file 1 [file biology-10-01069-s001.zip › biology-1393253-supplementary.pdf]

Supplementary

Table S1. Full electronic search in MEDLINE database through July 31st, 2020.

Research	Query	Items Found
1	Neupogen	906
2	filgrastim	891
3	granulocyte colony-stimulating factor	4823
4	G-CSF	5202
5	GCSF	2183
6	recombinant human granulocyte colony stimulating factor	1801
7	rhG-CSF	226
8	rhGCSF	230
9	r-met-HUG-CSF	2
10	r-metHuG-CSF	898
11	hematopoietic growth factor	6851
12	1 or 2	906
13	3 or 4 or 5 or 6	3831
14	7 or 8	233
15	9 or 10	39
16	12 or 13 or 14 or 15 or 11	7973

Table S2. Key characteristics of included studies of Filgrastim versus Placebo/ No treatment.

Author; Year	Study Design	Cancer Type	Cancer Stage	Country	Patients (N)	Patient age	Sex (M/F)	Chemotherapy regimen	Treatment Group	Intervention dose; Route
**Chemotherapy-Induced Neutropenia (CIN)**										
Chen *et al*; 2017	Observational; NA; NA	Colorectal cancer	NA	China	N= 100<br>Filgrastim: n = 50<br>Placebo: n= 50	Median age (range)<br>Filgrastim: 57 (25-77)<br>Placebo: 58 (27-75)	Filgrastim: 30/20<br>Placebo: 29/21	NA	Filgrastim vs. Placebo	150 µg/day; S.C
Altwairgi *et al*; 2013	Observational; NA; NA	Breast Cancer	NA	Canada	N = 239<br>PP G-CSF (filgrastim or pegfilgrastim) = 145<br>No PP G-CSF (secondary G-CSF or no G-CSF) = 94	Median (range)<br>55 (32–80)	0/239	Trastuzumab and docetaxel	PP G-CSF (filgrastim or pegfilgrastim) vs. No PP G-CSF (secondary G-CSF or no G-CSF)	NA
Hershman *et al*; 2009	Observational; NA; NA	Breast Cancer, lung cancer, ovarian cancer, or colon cancer, or lymphoma	NA	USA	N = 3123<br>PP G-CSF (filgrastim or pegfilgrastim) = 822<br>No PP G-CSF (delayed filgrastim or pegfilgrastim = 1523 or no G-CSF = 778)	<65 years: 61% in PP vs 58% in no G-CSF<br>>65 years: 38% in PP vs 41.2% in no G-CSF	PP G-CSF (filgrastim or pegfilgrastim) = 212/610<br>No PP G-CSF (delayed filgrastim or pegfilgrastim = 320/1203 or no G-CSF = 257/521)	Various	PP G-CSF (filgrastim or pegfilgrastim) vs. No PP G-CSF (delayed filgrastim or pegfilgrastim	NA

Blayney *et al*; 2005	NRCT; Phase I; NA	NSCLC, NHL	IIIA, IIIB, IV	USA	N= 104<br>NSCLC (n= 55):<br>Filgrastim: n = 46<br>No filgrastim: n= 9<br>NHL (n= 49):<br>Filgrastim: n = 44<br>No filgrastim: n= 5	Median (range)<br>NSCLC (n= 55): 59 (39-79)<br>NHL (n= 49): 53 (28-73)	NSCLC<br>Filgrastim: 29/17<br>NHL<br>Filgrastim: 26/18	NSCLC: etoposide, cisplatin<br><br>NHL: CHOP	Filgrastim vs. No Filgrastim	NSCLC trial: 5µg/kg/day<br><br>NHL trial: 5 µg/kg/day;
Crawford *et al*; 2005	RCT; Phase III; DB	SCLC	Limited/ extensive	USA	N= 231<br>Filgrastim: n = 111<br>Placebo: n= 120	Mean age (range ± SD)<br>Filgrastim: 61.2 (31-78 ±9.7)<br>Placebo: 62 (33- 80 ±8.6)	Filgrastim: 70/41<br>Placebo: 79/41	CDE	Filgrastim vs. Placebo	5 µg/kg/day; S.C.
Doorduijn *et al*; 2003	RCT; Phase III; NA	Aggressive NHL	II/ III/ IV	Dutch, The Netherland, Belgium	N= 389<br>Filgrastim: n = 197<br>Placebo: n= 192	Mean age ± SD<br>Filgrastim: 73 ± 5<br>Placebo: 73 ± 5	Filgrastim: 107/90<br>Placebo: 109/83	CHOP	Filgrastim vs. Placebo	300 µg/day; S.C.
Osby *et al;* 2003	RCT; NA; OL	Aggressive NHL	II/ III/ IV	Sweden, Norway, Denmark, Finland	N= 455<br>Filgrastim: n = 226<br>Placebo: n= 229	Median age 71 (60-86)	NA	CHOP; CNOP	Filgrastim vs. Placebo	5 µg/kg/day; S.C.
Papaldo *et al;* 2003	RCT; NA;OL	Breast Cancer	I/II	Italy	N= 503<br>Filgrastim: n = 254<br>No Filgrastim: n= 249	Median (range) 45 (25-65)	NA	AC	Filgrastim vs. No Filgrastim	300- 480 µg/day; S.C.

Gilad *et al;* 1999	Observational; NA; NA	Solid tumors or lymphoma	NA	Israel	N= 209 (1079 cycles)<br>PP filgrastim = 66<br>No PP= 1013	Median (range) 55 (19-88)	Filgrastim: 10/55<br>No Filgrastim: 258/755	Various	PP filgrastim vs. No PP filgrastim	3-5 μm/kg of body wt.; S.C.
Fossa *et al*; 1998	RCT; Phase III; OL	GCM	IV	UK, Norway, Hungary, The Netherland, Belgium	N= 259<br>Filgrastim: n = 129<br>No Filgrastim: n= 130	Median age 28 (15-65)	NA	BEP/EP	Filgrastim vs. No Filgrastim	5 μg/kg/day; S.C.
Larson *et al*; 1998	RCT; NA; OL	ALL	NA	USA	N= 198<br>Filgrastim: n = 102<br>Placebo: n= 96	Median (range)<br>35 (16-79)	NA	Intensive remission induction chemotherapy	Filgrastim vs. Placebo	5 μg/kg/day; S.C.
Michon *et al;* 1998	RCT; Phase II; OL	Metastatic Neuroblastoma	IV	France	N= 59<br>Filgrastim: n = 31<br>Placebo: n= 28	median (range)<br>Filgrastim: 3 (1-10)<br>Placebo: 3 (1-13)	Filgrastim: 24/7<br>Placebo: 18/10	VDC; EP	Filgrastim vs. Placebo	5 μg/kg/day; S.C.
Geissler *et al;* 1997	RCT; Phase III; NA	ALL	I,II,III,IV	Australia	N= 51<br>Filgrastim: n = 25<br>Placebo: n= 26	median (range)<br>Filgrastim:36.4 (17-75)<br>Placebo: 41.7 (16-79)	NA	DVAP	Filgrastim vs. Placebo	5 μg/kg/day; S.C.
Pui *et al;* 1997	RCT; NA; DB	ALL	NA	USA	N= 148<br>Filgrastim: n = 73<br>Placebo: n= 75	median (range)<br>Filgrastim:5.8 (0.2-17.9)<br>Placebo: 5.7 (1.0-16.9)	Filgrastim: 40/33<br>Placebo: 42/33	Remission induction therapy	Filgrastim vs. Placebo	10 μg /kg/day; S.C.

Zinzani *et al;* 1997	RCT; OL	Aggressive NHL	II/ III/ IV	Italy	N= 149<br>Filgrastim: n = 77<br>No filgrastim: n= 72	median (range)<br>Filgrastim: 69 (60-82)<br>No filgrastim: 70 (60-80)	NA	VNCOP-B	Filgrastim vs. No Filgrastim	5 mg/kg/day; S.C.
Ottmann; 1995	Observational; Phase III, OL	ALL	NA	Germany	N= 76<br>Filgrastim: n = 37<br>No filgrastim: n= 39	median (range)<br>Filgrastim: 27 (16-65)<br>No filgrastim: 30 (16-58)	Filgrastim: 26/11<br>Placebo: 25/14	Induction chemotherapy	Filgrastim vs. No Filgrastim	5 μg/kg/day; S.C.
Maher *et al*; 1994	RCT; Phase III; DB	ALL/Lymphoma	NA	Australia	N= 216<br>Filgrastim: n = 109<br>Placebo: n= 107	median (range)<br>Filgrastim: 48 (16-81)<br>Placebo: 51 (17-85)	Filgrastim: 54/55<br>Placebo: 49/58	Antibiotic therapy	Filgrastim vs. Placebo	12 μg/kg/day; S.C.
Gebbia *et al*; 1993	Observational ; NA, NA	Breast Cancer/SCLC/HNC/HC/GC	Advanced	Italy	N= 86<br>Filgrastim: n = 43<br>Placebo: n= 43	mean age (range)<br>Filgrastim: 56 (41-66)<br>Placebo: 58 (38-65)	Filgrastim: 16/27<br>Placebo: 15/28	Multiple	Filgrastim vs. Placebo	5 μg/kg/day; S.C.
Trillet-Lenoir *et al*; 1993	RCT; Phase III; DB	SCLC	I/ II/ III/ IV	Europe (13 centers)	N= 129<br>Filgrastim: n = 65<br>Placebo: n= 64	median<br>Filgrastim: 58<br>Placebo: 60	Filgrastim: 45/20<br>Placebo: 44/20	CDE	Filgrastim vs. Placebo	230 μg/m²/day; S.C
Crawford *et al*; 1991	RCT; Phase III; DB	SCLC	I/ II/ III/ IV	USA	N= 199<br>Filgrastim: n = 95<br>Placebo: n= 104	median (range)<br>Filgrastim: 62 (31-78)<br>Placebo: 63 (31-80)	Filgrastim: 65/35<br>Placebo: 63/37	CDE	Filgrastim vs. Placebo	230 μg/m²/day; S.C
**Acute Myeloid Leukemia (AML)**										
Beksac *et al*; 2011	RCT; Phase III; NA	AML	NA	Turkey	N= 260<br>Filgrastim: n = 123<br>No Filgrastim: n=137	Median (SD)<br>Filgrastim: 38.9 (13.5)<br>No Filgrastim: 38.3 (14.0)	Filgrastim: 74/49<br>No Filgrastim: 74/63	De novo AML induction therapy cytarabine and	Filgrastim vs. No Filgrastim	5 μg/kg/ day; I.V.

								idarubicin		
Heil *et al*; 2006	RCT; NA; DB	AML	NA	Germany, Spain, Austria, UK, Belgium, Portugal, Sweden, Italy, Australia	N= 521<br>Filgrastim: n = 259<br>Placebo: n= 262	Median (range)<br>Filgrastim: 54 (16-89)<br>Placebo: 54 (16-88)	Filgrastim: 141/118<br>Placebo: 142/120	Standard induction and consolidation chemotherapy	Filgrastim vs. Placebo	5 µg/kg/ day; S.C.
Usuki *et al*; 2002	RCT; NA; NA	AML	NA	Japan	N= 245<br>Filgrastim: n = 120<br>No Filgrastim: n=125	Mean (range)<br>Filgrastim: 48.5 (15-75)<br>No Filgrastim: 49.7 (15- 87)	Filgrastim: 80/40<br>No Filgrastim: 78/47	Induction chemotherapy	Filgrastim vs. No Filgrastim	200 µg/m²/day; S.C.
Harousseau *et al*; 2000	RCT; NA; NA	AML	NA	France	N= 194<br>Filgrastim: n = 100<br>No Filgrastim: n=94	Median (range):<br>Filgrastim: 47.5 (16-60)<br>No Filgrastim: 45 (15-60)	Filgrastim: 49/51<br>No Filgrastim: 47/47	Consolidation with either high dose cytarabine plus mitoxantrone or	Filgrastim vs. No Filgrastim	5 µg/kg/ day; S.C.

								amsacrine plus etoposide		
Godwin *et al*; 1998	RCT; Phase III, DB	AML	NA	USA	N= 211<br>Filgrastim: n = 106<br>Placebo: n=105	Median (range)<br>68 (56-88)	Filgrastim: 56/50<br>Placebo: 66/39	Induction with cytarabine, daunorubicin	Filgrastim vs. Placebo	400 µg/m²/day; I.V.
Heil *et al*; 1997	RCT; Phase III; DB	AML	NA	Germany, Spain, Belgium, Portugal, Sweden, UK, Italy, Australia	N= 521<br>Filgrastim: n = 259<br>No Filgrastim: n=262	Median (range)<br>Filgrastim: 54 (16-89)<br>No Filgrastim: 54 (16-88)	NA	Induction therapy with DCE	Filgrastim vs. No Filgrastim	5 µg/kg/ day; S.C.
Moore *et al*; 1997	NRCT; Phase II; NA	AML	NA	USA	N= 123<br>Filgrastim: n = 61<br>No Filgrastim: n=62	Median (range)<br>41 (16-59)	113/136	Consolidation with diaziquone, mitoxantrone	Filgrastim vs. No Filgrastim	5 µg/kg/ day
**Severe Chronic Neutropenia (SCN)**										

Yilmaz *et al*; 2007	Observational; NA; NA	SCN	NA	Turkey	N= 39<br>Filgrastim: n= 16<br>No filgrastim: n= 23	Median (range):<br>15 months (3 months to 17 years)	NA	NA	Filgrastim vs. No Filgrastim	5 µg/kg/ day
Dale *et al*; 1993	RCT; Phase III; NA	SCN	NA	USA	N= 123<br>Filgrastim: n= 63<br>4 month observation + filgrastim: n= 60	Median (range)<br>12.1 (0.6- 75.7)	Filgrastim: 29/34<br>4 month observation + filgrastim: 27/33	NA	Filgrastim vs. 4 month observation + filgrastim	Idiopathic neutropenia: 3.45 µg/kg/ day BID; S.C.<br>Cyclic neutropenia: 5.75 µg/kg/day BID; S.C.<br>Congenital neutropenia: 11.50 µg/kg/day BID; S.C.
**Bone Marrow Transplantation (BMT)**										
Gertz *et al*; 2011	Observational; NA; NA	MM	NA	USA	N= 664<br>Filgrastim: n= 498<br>No filgrastim: n= 166	Median (range):<br>Filgrastim: 59.8 (53.0-65.4)<br>No filgrastim: 61 (53.9-66.8)	Filgrastim: 290/208<br>No filgrastim: 102/64	NA	Filgrastim vs. No Filgrastim	5 µg/kg/ day
Gonzalez-Vicent *et al*; 2004	RCT; NA; NA	Acute leukemia, lymphoma, solid tumor	NA	Spain	N= 117<br>Filgrastim: n= 51<br>No filgrastim: n= 66	Median (range):<br>Filgrastim: 8 (1-18)<br>No filgrastim: 8 (1-18)	Filgrastim: 32/19<br>No filgrastim: 45/21	NA	Filgrastim vs. No Filgrastim	10 µg/kg/ day; S.C.
Damiani *et al*; 1997	Observational; NA; NA	NHL or HL	NA	Italy	N= 55<br>Filgrastim primed Bone marrow: n= 36	Median (range)	Filgrastim primed Bone marrow: 23/13	NA	Filgrastim primed Bone marrow	16 µg/kg/ day; S.C.

					Filgrastim primed peripheral blood : n= 19	Filgrastim primed Bone marrow: 32 (16-56)<br>Filgrastim primed peripheral blood : 41 (22-55)	Filgrastim primed peripheral blood : 10/09		vs.Filgrastim primed peripheral blood	
Stahel *et al*; 1997	RCT; NA; OL	NHL or HL	NA	Switzerland	N= 86<br>Filgrastim 5 µg/kg/ day:<br>n= 44<br>Filgrastim 10 µg/kg/ day:<br>n= 42	Median (range)<br>Filgrastim 5 µg/kg/ day:<br>37.5 (16-58)<br>Filgrastim 10 µg/kg/ day:<br>32.5 (16-56)	Filgrastim 5 µg/kg/ day:<br>27/17<br>Filgrastim 10 µg/kg/ day:<br>26/16	NA	Filgrastim 5 µg/kg/ day vs. Filgrastim 10 µg/kg/ day	5 or 10 µg/kg/ day; S.C.

Abbreviations: AC= Doxorubicin [or pirarubicin or epirubicin], cyclophosphamide; **ALL**= Acute lymphoblastic leukemia; **AML**= Acute myeloid leukemia; **BD**= Bortezomib and dexamethasone; **BEP/EP**= Cisplatin, etoposide, bleomycin; **BHAC-DM/ BHAC-DMP=** behenoyl-cytosine arabinoside, daunorubicin and 6-mercaptopurine, prednisolone; **CDE** = cyclophosphamide, doxorubicin, etoposide; **CHASE (R)**= cyclophosphamide, cytarabine, etoposide and dexamethasone± rituximab; **CIN**= Chemotherapy-Induced Neutropenia; **CHOP** = Cyclophosphamide, doxorubicin, vincristine, prednisolone; **DB** = double-blind; **DCE**= Daunorubicin, cytarabine and etoposide; **DD**= doxorubicin and docetaxel; **DLBCL**= Diffuse large B-cell lymphomas; **DVAP**= daunorubicin, vincristine, L-asparaginase and prednisone; **ET**= docetaxel, cyclophosphamide; **FEC-D**= fluorouracil, epirubicin, cyclophosphamide and docetaxel; **GCM**= Germ cell malignancy; **GCSF=** Granulocyte colony stimulating factor;**HL**= Hodgkin's Lymphoma; **IC**= Idarubicin, cytarabine; **IE**= etoposide and ifosfamide; **I.V.** = intravenously; **MM**= Multiple myeloma; **MMM**= mitomycin, mitoxantrone, methotrexate; **MOPP**= Mustine, vincristine (Oncovin), procarbazine and prednisolone; **MOPP/EVAC**= Multi-drug chemotherapy with MOPP alternating every 2 weeks with etoposide, vinblastine, Adriamycin and prednisolone; **NA**= Not available; **NHL**= non-Hodgkin lymphoma; **NRCT**= Non-randomized clinical trial; **NSCLC**= Non-small cell lung cancer; **OL**= Open Label; **PA**= Paclitaxel, doxorubicin [or pirarubicin or epirubicin];**PC**= Paclitaxel, cisplatin; **PP**= Primary Prophylactic; **RCT**= Randomized clinical trial; **r-metHuG-CSF=** Recombinant methionyl human granulocyte colony-stimulating factor; **S.C**.= subcutaneously; **SCLC**= Small-cell lung carcinoma; **SD**: Standard deviation; **TAC** = doxorubicin, cyclophosphamide, docetaxel; **TC**= epirubicin, docetaxel; **TEC**= Docetaxel, Epirubicin, cyclophosphamide; **VDC**= vincristine, doxorubicin, cyclophosphamide; **VAPEC-B=** vincristine, doxorubicin (Adriamycin), prednisone, etoposide, cyclophosphamide, bleomycin; **VNCOP-B=** cyclophosphamide, mitoxantrone, vincristine, etoposide, bleomycin, and prednisone.

Table S3. Key characteristics of included studies of Filgrastim versus Pegfilgrastim.

Author; Year	Study Design	Cancer Type	Cancer Stage	Country	Patients (N)	Patient age	Sex (M/F)	Chemotherapy regimen	Treatment Group	Intervention dose; Route
**Chemotherapy-Induced Neutropenia (CIN)**										
Fengrui *et al*; 2019	RCT; Phase III; OL	Breast Cancer	NA	China	N= 339<br>Filgrastim: n = 113<br>MPEG 110 µg/kg: n= 113<br>MPEG 6mg: n= 113	Mean age ± SD<br>Filgrastim: 47.37±8.60<br>MPEG 110 µg/kg: 48.21±8.55<br>MPEG 6mg: 48.03±9.01	Filgrastim: 0/110<br>MPEG 110 µg/kg: 0/111<br>MPEG 6mg: 0/110	TC; AC	Filgrastim and Pegfilgrastim (mecapegfilgrastim (MPEG))	5 µg/kg/ day vs. 100 µg/kg or 6 mg
Kubo *et al*; 2016	RCT; Phase III; DB	NHL or HL	I,II,III,IV	Japan	N= 111<br>Filgrastim: n = 56<br>Pegfilgrastim: n= 55	Median (range)<br>Filgrastim: 60.5 (24-79)<br>Pegfilgrastim: 61 (28-74)	Filgrastim: 31/23<br>Pegfilgrastim: 35/18	CHASE (R)	Filgrastim and Pegfilgrastim	50 µg/m²/day vs. 3.6 mg/cycle
Park *et al*; 2017	RCT; Phase III; OL	Breast Cancer	III	South Korea	N= 74<br>Filgrastim: n = 38<br>DA 3031: n= 36	Median (range)<br>Filgrastim: 48 (30,67)<br>DA 3031: 48 (33,66)	0/74	TAC	Filgrastim and peg-G-CSF	100 µg/kg/ day vs. 6 mg/cycle
Filon *et al*; 2015	RCT; Phase III; DB	Breast Cancer	II/ III/ IV	Russia	N= 135<br>Filgrastim: n = 45<br>EMPEG 6 mg/kg: n= 45<br>EMPEG 7.5 mg: n= 45	Age range 18-65; mean age 50.2	0/135	DD	Filgrastim and Empegfilgrastim (EMPEG)	5 µg/kg/day vs. 6 mg/cycle or 7.5 mg

Zhang *et al*; 2015	RCT; Phase II; OL	Breast Cancer	NA	China	N= 171<br>Filgrastim: n = 43<br>PEG 60 μg /kg: n= 43<br>PEG 100 μg/kg: n= 43<br>PEG 120 μg/kg: n= 42	Mean age (SD)<br>Filgrastim: n = 47.35 (8.14)<br>PEG 60 μg /kg: 47.03 (7.66)<br>PEG 100 μg/kg: 48.18 (8.09)<br>PEG 120 μg/kg: 46.71 (6.80)	0/171	TAC	Filgrastim and Pegfilgrastim	5 μg/kg/ day vs. 60 or 80 or 100 μg/kg/day
Park *et al*; 2013	RCT; Phase II; OL	Breast Cancer	II/ III	South Korea	N= 61<br>Filgrastim: n = 21<br>DA-3031 3.6 mg: n= 20<br>DA-3031 6 mg : n= 20	Median (range)<br>Filgrastim: 47 (29-56)<br>DA-3031 3.6 mg: 43 (34-54)<br>DA-3031 6 mg : 46 (34,67)	0/61	TAC	Filgrastim and Pegfilgrastim (DA 3031)	100 μg/kg/ day vs. 3.6 or 6 mg/cycle
Salafet *et al*; 2013	RCT; Phase II; OL	Breast Cancer	NA	Russia	N= 60<br>Filgrastim: n = 19<br>BCD 017 3 mg: n= 21<br>BCD 017 6 mg: n= 20	NA	0/60	DD	Filgrastim and BCD 017 Empegfilgrastim (EPEG)	5 μg/kg/day vs.3 mg or 6 mg/cycle
Green *et al*; 2003	RCT; phase III; DB	Breast Cancer	NA	Australia	N= 152<br>Filgrastim : 75<br>Pegfilgrastim: 77	Mean age (SD):<br>Filgrastim : 52.8 (11.5)<br>Pegfilgrastim: 52.1 (9.2)	Filgrastim: 74/1<br>Pegfilgrastim: 77/0	DD	Filgrastim and Pegfilgrastim	5 μg/kg/ day vs. 6 mg/day

Grigg *et al*; 2003	RCT; phase II; OL	NHL	NA	Europe, Australia	N= 50<br>Filgrastim : 22<br>Pegfilgrastim: 27	Mean age (SD):<br>Filgrastim : 67.5 (5.7)<br>Pegfilgrastim: 69.6 (5.8)	Filgrastim: 14/8<br>Pegfilgrastim: 9/18	CHOP	Filgrastim and Pegfilgrastim	5 µg/kg/ day vs. 60 µg/kg/day and 100 µg/kg/day
Vose *et al*; 2003	RCT; Phase II; OL	Relapsed or refractory HL or NHL	I/ II/ III/ IV	USA	N= 60<br>Filgrastim : 31<br>Pegfilgrastim: 29	Mean age (SD):<br>Filgrastim : 48.4 (15.9)<br>Pegfilgrastim: 50.6 (13.9)	Filgrastim: 17/14<br>Pegfilgrastim: 19/10	CHOP	Filgrastim and Pegfilgrastim	5 µg/kg/ day vs. 100 µg/kg/ day
Holmes *et al*; 2002	RCT; Phase III; DB	Breast Cancer	II/ III/ IV	USA	N= 296<br>Filgrastim : 147<br>Pegfilgrastim: 149	Mean age ±SD:<br>Filgrastim : 51.9 ± 11.1<br>Pegfilgrastim: 50.9 ± 11.7	Filgrastim: 1/146<br>Pegfilgrastim: 2/147	DD	Filgrastim and Pegfilgrastim	5 µg/kg/ day vs. 100 µg/kg/ day
Holmes *et al*; 2002	RCT; Phase II; DB	Breast Cancer	II/ III/ IV	USA	N= 125<br>Filgrastim: n= 25<br>PEG 30 µg /kg: n= 19<br>PEG 60 µg/kg: n= 60<br>PEG 100 µg/kg: n= 46	Mean age (SD):<br>Filgrastim: 50 (9)<br>PEG 30 µg /kg: 51 (13)<br>PEG 60 µg/kg: 51 (11)<br>PEG 100 µg/kg: 49 (11)	0/125	DD	Filgrastim and Pegfilgrastim	5 µg/kg/ day vs. 30 or 60 or 100 µg/kg/ day
**Acute Myeloid Leukemia (AML)**										
Sierra *et al*; 2008	RCT; Phase II; DB	AML	NA	Spain	N= 83<br>Filgrastim: n= 41<br>Pegfilgrastim: n= 42	median (range)<br>Filgrastim: 51 (18-74)	Filgrastim: 17/24<br>Pegfilgrastim: 22/20	IC	Filgrastim and Pegfilgrastim	5 µg/kg/day vs 6 mg/ cycle

Pegfilgrastim: 54 (19-79)

Abbreviations: AC= Doxorubicin [or pirarubicin or epirubicin], cyclophosphamide; **AML**= Acute myeloid leukemia; **BCD**= Bortezomib, cyclophosphamide and dexamethasone; **CHASE (R)**= cyclophosphamide, cytarabine, etoposide and dexamethasone± rituximab; **CIN**= Chemotherapy-Induced Neutropenia; **CHOP** = Cyclophosphamide, doxorubicin, vincristine, prednisolone; **DB** = double-blind; **DD**= doxorubicin and docetaxel; **EMPEG:** Empegfilgrastim; **GCSF=** Granulocyte colony stimulating factor;**HL**= Hodgkin's Lymphoma; **IC**= Idarubicin, cytarabine; **MPEG**= Mecapegfilgrastim; **NA**= Not available; **NHL**= non-Hodgkin lymphoma; **OL**= Open Label; **PEG**: Pegfilgrastim;**RCT**= Randomized clinical trial; **SD**: Standard deviation; **TAC** = doxorubicin, cyclophosphamide, docetaxel; **TC**= epirubicin, docetaxel.

Table S4. Key characteristics of included studies of Filgrastim versus Biosimilar Filgrastim.

Author; Year	Study Design	Cancer Type	Cancer Stage	Country	Patients (N)	Patient age	Sex (M/F)	Chemotherapy regimen	Treatment Group	Intervention dose; Route
**Chemotherapy-Induced Neutropenia (CIN)**										
Blackwell *et al*; 2018	RCT; phase III; DB	Breast Cancer	I/II/III	USA	N= 213<br>Filgrastim: n = 51<br>Switched biosimilar: n= 109<br>EP2006: n= 53	median age:<br>Filgrastim: 46.5<br>Switched: 50.0	0/213	TAC	EP2006 and filgrastim innovator	5 µg/kg/day vs. 5 µg/kg/day
Hegg *et al*; 2016	RCT; Phase III; OL	Breast Cancer	II/ III/ IV	Brazil	N= 217<br>Filgrastim: n= 108<br>Biosimilar filgrastim: n= 109	mean age ± SD<br>Filgrastim:49.04 ±11.24<br>Biosimilar filgrastim: 51.36±9.85	0/217	TAC; DD	Filgrastim and biosimilar filgrastim (Fiprima®)	5 µg/kg/ day vs.5 µg/kg/ day
Blackwell *et al*; 2015	RCT; phase III; DB	Breast Cancer	I/II/III	USA	N= 214<br>Filgrastim: n= 107<br>EP 2006: n= 107	mean age (SD):<br>Filgrastim: 48.4 (11.02)<br>EP 2006: 49.5 (11.52)	0/218	TAC	EP2006 and filgrastim innovator	5 µg/kg/day vs. 5 µg/kg/day

Waller *et al*; 2010	RCT; Phase III; DB	Breast Cancer	NA	Germany	N= 278<br>Filgrastim: n= 95<br>Biosimilar filgrastim: n= 183	mean age (SD):<br>Filgrastim: 50 (8.94)<br>Biosimilar filgrastim: 49.5 (8.78)	Filgrastim: 0/95<br>Biosimilar filgrastim: 0/183	DD	Filgrastim and biosimilar filgrastim (Hospira)	5 µg/kg/ day vs.5 µg/kg/ day
Engert *et al*; 2009	RCT; Phase III; NA	Aggressive NHL	NA	Germany	N= 92<br>Filgrastim: n= 29<br>XM02: n= 63	Median (range):<br>Filgrastim: 54 (18-83)<br>XM02: 57 (33-83)	Filgrastim: n= 17/12<br>XM02: n= 31/32	CHOP	Filgrastim and biosimilar filgrastim XM02	5 µg/kg/day vs. 5 µg/kg/day
del Giglio *et al*; 2008	RCT; Phase III; SB	Breast Cancer	II/ III/ IV	Brazil, Belarus, Lithuania, Romania, Germany, Chile, South Africa, Russia, Poland	N= 348<br>Filgrastim: n = 136<br>XM02: n= 140<br>Placebo/ XM02: n= 72	median (range)<br>Filgrastim: 51 (28-74)<br>XM02: 51 (25-75)<br>Placebo/ XM02: 48 (28-74)	Filgrastim: 1/135<br>XM02: 1/139<br>Placebo/ XM02: 0/72	DD	Filgrastim and biosimilar filgrastim XM02 and placebo	Filgrastim and XM02:<br>5 µg/kg/day; S.C.
**Peripheral Blood Collection by Leukapheresis (PBCL)**										
Sivgin *et al*; 2016	Observational ; NA; NA	AML; ALL; aplastic anaemia; lymphomas	NA	Turkey	N= 243<br>Filgrastim: n= 201<br>Filgrastim biosimilar (Leucostim): n= 14<br>Lenograstim: n= 28	Mean age (donor)<br>Filgrastim: 31.20±12.92<br>Filgrastim biosimilar (Leucostim): 34.07 ± 11.67	Filgrastim: 107/94<br>Filgrastim biosimilar (Leucostim): 8/6<br>Lenograstim: 18/10	alloHSCT	Filgrastim vs. Filgrastim biosimilar (Leucostim) vs. Lenograstim	11µg/kg/day vs. 10.35 µg/kg/day vs. 11µg/kg/day

						Lenograstim: 29.21 ± 11.05<br><br>Mean age (patient)<br>Filgrastim: 31.12 ±11.35 Filgrastim biosimilar (Leucostim): 32.43 ± 9.94<br>Lenograstim: 25.75 ± 8.72				
Skopec *et al*; 2016	Observational; NA; NA	MM	NA	Europe	N= 39<br>Filgrastim: n= 20<br>Pegfilgrastim: n= 19	Median (range)<br>Filgrastim: 60 (35-69)<br>Pegfilgrastim: 64 (51-71)	Filgrastim: 11/9<br>Pegfilgrastim: 9/10	alloHSCT	Filgrastim and Pegfilgrastim	10 µg/kg/day; S.C.
Yoshimura *et al*; 2017	Observational; NA; NA	Malignant Lymphoma and MM	NA	Japan	N= 69<br>Filgrastim: n= 34<br>Filgrastim biosimilar (XM02): n= 12<br>Lenograstim: n= 23	Median (range)<br>Filgrastim: 58 (30-72)<br>Filgrastim biosimilar (XM02): 55 (23-66)<br>Lenograstim: 56 (28-69)	Filgrastim: 17/17<br>Filgrastim biosimilar (XM02): 7/5<br>Lenograstim: 15/18	NA	Filgrastim vs. Filgrastim biosimilar (XM02) vs. Lenograstim	5 µg/kg/ day; S.C.

Abbreviations: alloHSCT= allogeneic hematopoietic stem cell transplantation; **CHOP** = Cyclophosphamide, doxorubicin, vincristine, prednisolone; **DB**= double-blind; **DD**= doxorubicin and docetaxel; MM= Multiple Myeloma; **NA**= Not available; **OL**= Open Label; **PBCL**= Peripheral Blood Collection by Leukapheresis; **RCT**= Randomized clinical trial; **SB**= single blind; **SD**: Standard deviation; **TAC**= doxorubicin, cyclophosphamide, docetaxel.

Table S5. Baseline and clinical patients' characteristics: (Filgrastim versus Placebo controlled trial/ no treatment).

	CIN (1)		AML (2)		SCN (3)	
	Filgrastim (n= 2837)	Placebo controlled/ No treatment (n= 3608)	Filgrastim (n= 1028)	Placebo controlled/ No treatment (n= 1047)	Filgrastim (n= 79)	Placebo controlled/ No treatment (n= 83)
Age (median)	56	55	49	47	6	6
Women, %	70	71	36	37	53	49

Abbreviations: CIN: Chemotherapy Induced Neutropenia; AML: Acute Myeloid Leukaemia; SCN: Severe chronic neutropenia.

Table S6. Baseline and clinical patients' characteristics (Filgrastim versus Pegfilgrastim).

	CIN (1)		AML (2)	
	Pegfilgrastim (n= 1023)	Filgrastim (n= 635)	Pegfilgrastim (n= 42)	Filgrastim (n= 41)
Age (median)	50	49	54	51
Women, %	72	81	47	58

Abbreviations: CIN: Chemotherapy Induced Neutropenia; AML: Acute Myeloid Leukaemia.

Table S7. Baseline and clinical patients' characteristics (Filgrastim versus Biosimilar Filgrastim).

	CIN (1)	
	Filgrastim (n= 526)	Biosimilar Filgrastim (n= 836)
Age (median)	50	50
Women, %	97	96

Abbreviations: CIN: Chemotherapy Induced Neutropenia.

Table S8. Type of Trial reported (Filgrastim versus Placebo controlled trial/ no treatment).

	CIN (1) (n=21)	AML (2) (n= 7)	SCN (3) (n= 2)	BMT (4) (n= 4)
**RCT**	14	6	1	2
**NRCT**	1	1	0	0
**Observational**	6	0	1	2

Abbreviations: AML: Acute Myeloid Leukaemia; CIN: Chemotherapy Induced Neutropenia; RCT: Randomized clinical trial; NRCT: Non-randomized clinical trial; SCN: Severe chronic neutropenia.

Table S9. Type of Trial reported (Filgrastim versus Pegfilgrastim).

	CIN (1) (n=12)	AML (2) (n= 1)
**RCT**	12	1
**NRCT**	0	0
**Observational**	0	0

Abbreviations: AML: Acute Myeloid Leukaemia; CIN: Chemotherapy Induced Neutropenia; RCT: Randomized clinical trial; NRCT: Non-randomized clinical trial.

Table S10. Type of Trial reported (Filgrastim versus Biosimilar Filgrastim).

	CIN (1) (n=6)	PBCL (2) (n= 3)
**RCT**	6	0
**NRCT**	0	0
**Observational**	0	3

Abbreviations: RCT: Randomized clinical trial; NRCT: Non-randomized clinical trial; PBCL: Peripheral Blood Collection by Leukapheresis.

Table S11. Key characteristics of studies that compared Filgrastim with Placebo or no treatment by indication and study type.

.			Efficacy and Effectiveness						Safety	
**Author; Year**	**Cancer Type**	**Patients (N)**	**Time to ANC or platelet recovery**	**Incidence/ Duration of FN**	**Incidence/ Duration of grade 3 or 4 neutropenia**	**Incidence of infection/ antibiotic use**	**Incidence/ Duration of Hospitalization**	**RDI, Dose Delays or dose reductions**	**Survival/ mortality**	**AE**
Chemotherapy-Induced Neutropenia (CIN)										
Chen *et al*; 2017	Colorectal cancer	N= 100 Filgrastim: n = 50 Placebo: n= 50	-	FN incidence 0% vs. 6%	Leukopenia Grade III/ IV: 36% vs. 72% P <0.05	NA	NA	NA	NA	AEs in treatment group: low-grade fever/chill (14%), fatigue (8%), nausea (6%), headache (12%).
Altwairgi *et al*; 2013	Breast Cancer	N = 239 PP G-CSF (filgrastim or pegfilgrastim) = 145 No PP G-CSF (secondary G-CSF or no G-CSF) = 94	-	FN incidence 14% vs. 31 % P = 0.002	NA	NA	NA	RDI (range) for pts who received the FEC/D regimen **98% (75%–117%) vs 95% (60%–100%)** P = 0.05 Achievement of RDI >85% for pts who received the FEC/D regimen 97% vs 92% P = 0.118 Dose delay 17% vs 27% P = 0.060 Dose reduction 19% vs 25% P = 0.28	NA	NA
Hershman *et al*; 2009	Breast Cancer, lung cancer, ovarian cancer, or	N = 3123 PP G-CSF (filgrastim or pegfilgrastim) = 822	-	PP G-CSF vs. no PP G-CSF 4.5% vs. 7.5% OR=0.49, 95% CI 0.34–0.71, p<0.001)	NA	NA	NA	NA	NA	NA

	colon cancer, or lymphoma	No PP G-CSF (delayed filgrastim or pegfilgrastim = 1523 or no G-CSF = 778)								
Blayney *et al*; 2005	NSCLC, NHL	N= 104<br>NSCLC (n= 55):<br>Filgrastim: n = 46<br>No filgrastim: n= 9<br>NHL (n= 49):<br>Filgrastim: n = 44<br>No filgrastim: n= 5	-	NA	Grade 3 and grade 4 neutropenia: 62% and 77% lower with filgrastim[b]<br>Median duration of grade 3 and grade 4 neutropenia: 81% and 94% lower[b]	NA	Mean (SD) days in hospital<br>NSCLC<br>12.8 (13.1) vs 15.1 (17.5)[b]<br>NHL<br>4.7 (8.4) vs 2.4 (3.3)[b]	NSCLC<br>Dose reduction 3% vs 12%[b]<br>Dose delay 12% vs 38%b<br>NHL<br>Dose reduction 12% vs 0%[b]<br>Dose delay 6% vs 12%[b]	NA	AEs reported not specific to G-CSF
Crawford *et al*; 2005	SCLC	N= 231<br>Filgrastim: n = 111<br>Placebo: n= 120	-	FN incidence 38% vs. 74%<br>P < 0.0001	Grade 4 neutropenia incidence in cycle 1 82% vs. 97%<br><br>Across all cycles grade 4 incidence 56% vs. 89%<br>P < 0.0001	NA	NA	Response rate 68% vs. 72%	NA	AEs reported not specific to G-CSF
Timmer-Bonte *et al*; 2005	SCLC	N= 175<br>Filgrastim: n = 90<br>Placebo: n= 85	-	FN incidence in cycle 1: 10% vs. 24%<br>P= 0.01	NA	Infection was 0.14 infections per patient vs. 0.21	Median duration of hospitalization 7days vs. 15days	NA	3% vs 6% mortality	Fever in cycle 1 was 12% vs 27%

				FN incidence 2 to 5 cycles: 11% vs 17%<br><br>Overall FN incidence 18% vs. 32%		infections per patient				
Doorduijn *et al*; 2003	Aggressive NHL	N= 389<br>Filgrastim: n = 197<br>Placebo: n= 192	-	FN incidence<br>72 pts (36.5%) vs 86 pts (44.8%)<br>Median (range) days<br>2 (1–14) vs 3 (1–32)<br>P = 0.04	NA	Infections<br>8% vs 12%<br>P = 0.004<br>Severe infections<br>3% vs 3%<br>P = 0.82<br>Median antibiotic use, days (range)<br>0 (0–126) vs 6 (0–180)<br>P = 0.006	Days (range) hospitalization<br>5 [0–157] vs 6 [0–111]<br>P = 0.40	Median (range) RDI 95.1% (39.4–110) vs 93.4% (47.7–109)<br>P = 0.12	OS at 5 years<br>24% vs 22%<br>P = 0.76	Grade 3/4 AEs<br>Neurotoxicity<br>13 (1%) vs 33 (3%)<br>Nausea/vomiting<br>15 (1%) vs 18 (2%)<br>Diarrhea<br>8 (1%) vs 2 (<1%)<br>Oral toxicity<br>2 (<1%) vs 4 (<1%)<br>Cardiac toxicity<br>9 (1%) vs 6 (1%)<br>Hemorrhage<br>NA vs 1 (<1%)<br>Liver toxicity<br>NA vs 1 (<1%)<br>Bone pain<br>3 (<1%) vs NA<br>Other<br>23 (2%) vs 30 (3%)
Osby *et al;* 2003	Aggressive NHL	N= 455<br>Filgrastim: n = 226<br>Placebo: n= 229	-	Granulocytopenic fever ($<0.5x10^9/L$)<br>CHOP arms<br>34% vs 50%<br>CNOP arms<br>32% vs 50%	Granulocytopenia ($<0.5x10^9/L$)<br>CHOP arms<br>55% vs 89%<br>CNOP arms<br>64% vs 86%	NA	Granulocytopenic fever requiring hospitalization ($0.5\times10^9/L$):<br>33% vs 50%<br>P = 0.001	RDI ≥90% during 8 courses<br>44% vs 34%<br>P < 0.05	OS rates<br>CHOP ± filgrastim<br>61% vs 51%<br>CNOP ± filgrastim<br>33% vs 33%	CHOP + filgrastim vs CHOP<br>Mucositis 5% vs 4%<br>GI toxicity 15% vs 10%<br>Alopecia 80% vs 81%<br>Cardiac toxicity 5% vs 3%<br>Musculoskeletal pain 10% vs 2%<br>CNOP + filgrastim vs CNOP

										Mucositis 3% vs 2%<br>GI toxicity 8% vs 5%<br>Alopecia 47 vs 41<br>Cardiac toxicity 3 vs 1
Papaldo *et al;* 2003	Breast Cancer	N= 503<br>Filgrastim: n = 254<br>No Filgrastim: n= 249	-	FN incidence 1.2% vs 6.6%<br>P = 0.004	Grade 3/4 Neutropenia 28.6% vs 81.6%<br>P < 0.00001	NA	NA	Dose reduction<br>1.4% vs 3.6%<br>P = 0.002<br>Dose delay<br>3.6% vs 10%<br>P < 0.0001<br>Dose intensity<br>98.1% vs 95.5% NS	5-year OS<br>80.6% vs 79.6%<br>NS<br><br>DFS<br>67.2% vs 72.9%<br>NS	Bone pain (grade 1 to 3)<br>42.5%<br>Fever (grade 1/2)<br>16.3%
Gilad *et al;* 1999	Solid tumors or lymphoma	N= 209 (1079 cycles)<br>Cycles with PP filgrastim = 66<br>Cycles with no PP= 1013	-	FN incidence 4.5% vs 3.7%<br>P = 0.441	NA	Infections<br>1.5% vs 1.0%<br>P = 0.781	Hospitalized pts<br>6.0% vs 4.5% P = 0.958	NA	Deaths:<br>1 pt vs 1 pt,<br>none from infectious complication	AEs during induction 1<br>Rash 3 vs 2<br>Musculoskeletal pain 2 vs 1
Fossa *et al;* 1998	GCM	N= 259<br>Filgrastim: n = 129<br>No Filgrastim: n= 130	-	FN incidence 20% vs 30%<br>P < 0.052	NA	Blood culture proven sepsis<br>6.3% vs 7.8%	NA	Received ≥6 chemotherapy cycles<br>86% vs 71%<br>P = 0.003<br><br>Dose intensity:<br>Significantly higher dose intensities with filgrastim	1-year survival<br>83% (78–91) vs 75% (67–82)<br><br>Death due to toxicities<br>5 vs 15	n, (%)<br>BEP/EP and BOP/VIP-B<br>WBC<br>Grade 3: 7 (11)/12 (18) and 23 (36)/24 (37)<br>Grade 4: 8 (13)/12 (18) and 8 (13)/32 (49)<br>Platelet count<br>Grade 3: 14 (22)/15 (23) and 4 (6)/10 (15)<br>Grade 4: 13 (21)/25 (38) and 6 (9)/22 (33)<br>Neutropenic fever<br>9 (14)/16 (25) and 8 (13)/30 (46)

										Blood culture proven sepsis<br>4 (6)/4 (6) and 3 (5)/7 (11)<br>Mucosal toxicity<br>Grade 3: 4 (6)/4 (6) and 2 (3)/3 (5)<br>Grade 4: 0 (0)/0 (0) and 0 (0)/3 (5)<br>Pulmonary toxicity<br>Grade 1/2: 16 (25)/ 11(17) and 10 (16)/9 (14)<br>Grade 3: 0 (0)/3 (5) and 2 (3)/1 (2)<br>Grade 4: 2 (3)/1 (2) and 0 (0)/3 (5)
Larson *et al*; 1998	ALL	N= 198<br>Filgrastim: n = 102<br>Placebo: n= 96	Median days (IQR) to ANC recovery (>1000/μL)<br>Course I<br>16 (15–18) vs 22 (19–29)<br>P < 0.001<br>Course IIA<br>20 (6–25) vs 29 (22–31)<br>P < 0.001<br>Course IIB<br>25 (15–32) vs 31 (27–39)<br>P < 0.001	NA	Median (IQ3) Neutropenia (ANC <1000/μL), days:<br>Course I<br>13 (10–16) vs 20 (15–27)<br>P < 0.001<br>Course IIA<br>5 (0–12) vs 13 (6–18)<br>P < 0.001<br>Course IIB<br>11 (4–17) vs 14 (10–25)<br>P = 0.001	Infections<br>78% vs 87% P = 0.13	Median (IQ3) hospital stay, days:<br>Course I<br>22 (18–29) vs 28 (22–33)<br>P = 0.02<br>Course IIA<br>7 (0–17) vs 3 (0–14)<br>P = 0.32<br>Course IIB<br>4 (0–21) vs 2 (0–15)<br>P = 0.17	NA	Estimated median overall survival after 4.7 years follow-up (years)<br>2.4 vs 1.8<br>P = 0.25<br>Died during induction, n (%):<br>All eNAolled pts<br>5 (5) vs 11 (11)	Grade 3/4/5 toxicity<br>Pain, 21% vs 14%, P = 0.026<br>All other AEs were not significantly different<br>Infection, 78% vs 87%<br>Malaise/fatigue<br>(PS >2), 16% vs 25%<br>Hemoglobin (<6.5 g/dL), 93% vs 86%<br>Hypofibrinogenemia (<0.5 x normal), 26% vs 18%<br>Bilirubin (>1.5 x normal), 44% vs 51%<br>Nausea, 23% vs 28%<br>Motor neuropathy, 18% vs 22%<br>WBC (<1000/μL), 98% vs 97%

			Median (IQR) days to platelet recovery (>50000/µL): Course I 16 (14–20) vs 19 (15–23) P = 0.003 Course IIA 20 (17–22) vs 20 (18–22) P = 0.53 Course IIB 24 (21–31) vs 22 (0–28) P = 0.03						All eligible pts 4 (4) vs 10 (11) Died in complete remission, n (%): 8 (8) vs 5 (5) Alive in continuous complete remission, n (%): 35 (41) vs 22 (31)	Platelets (<25000/µL), 97% vs 95% Hyperglycemia (>250 mg/dL), 33% vs 35% Transaminases (>5 x normal), 35% vs 35%
Michon *et al;* 1998	Metastatic Neuroblastoma	N= 59 Filgrastim: n = 31 Placebo: n= 28	-	FN Incidence CADO 42% vs. 57% P = 0.24 CDDP 16% vs. 36% P = 0.08 CADO 26% vs. 44% P = 0.14 CDDP 3% vs. 19% P = 0.06	NA	NA	Median hospital stay 20 days vs. 28 days	NA	Deaths 1 patient vs. 1 Patient	Median duration of fever 4 days vs. 6 days P = 0.12
Geissler *et al;* 1997	ALL	N= 51 Filgrastim: n = 25 Placebo: n= 26	median times to recovery to ANC ≥	FN Incidence 12% vs. 42%	NA	Infections 40% vs. 77% Patients with infections	NA	NA	Death 1 patient vs. 2	Hyperbilirubinemia 15 vs. 11 Hypofibrinogenemia 11 vs. 11

			1000 and ≥ 500/μL were significantly shorter in the G-CSF group as compared with the controls (ANC ≥ 1,000: 16 v 26 days, P < 0.0005, logrank test; ANC ≥ 500: 16 v 24 days, P < 0.005)			Septicemia 2 vs. 8<br>Bacteremia 2 vs. 3<br>FUO 1 vs. 4<br>Pneumonia 1 vs. 3<br>Oral infection 3 vs. 5<br>Skin infection 1 vs. 1<br>Herpes 4 vs. 5<br>Otitis 0 v. 1				Diarrhea 1 vs. 5<br>Constipation 1 vs. 2<br>Nausea/vomiting 2 vs. 0<br>Psychiatric symptoms 1 vs. 0<br>Peripheral neuropathy 2 vs. 0<br>Hyperglycemia 1 vs. 2<br>Gut perforation 1 vs. 1<br>Pneumothorax 1 vs. 0<br>Rash 0 vs. 1
Pui *et al*; 1997	ALL	N= 148<br>Filgrastim: n = 73<br>Placebo: n= 75	ANC recovery<br>Median days (range) for recovery to $0.5x10^9$<br>5.3 vs 12.7<br><br>Platelets recovery ($x10^{-3}$/ mm^3)<br>14 (2–330) vs 18 (3–120)<br><75000/mm^3	Median (range) days with fever 2 (0–36) vs 2 (0–27)	NA	All infections<br>12 pts (16%) vs 27 (36%)<br>P = 0.009<br><br>Grade 3/4 infections<br>5 pts (7%) vs 6 pts (8%)<br><br>IV antibiotics use<br>42 pts vs 51 pts<br><br>Median days (range) duration of	Incidence of FN-related hospitalization<br>42 pts (58%) vs 52 pts (68%)<br>P = 0.23<br><br>Median days (range) duration hospital stay for FN<br>6 (1–37) vs 10 (1–30)<br>P = 0.011	NA	EFS at 3 years, 83% (both groups)	Grade ¾ Pneumonia 3 vs 2<br>Bacteremia 1 vs 3<br>Disseminated fungal infection 0 vs 1<br>Typhlitis 1 vs 0<br>AML incidence 5.1% vs 3.9%<br>P = 0.39

			8.9 vs 8.3			IV antibiotic use 6 (2–36) vs 9 (2–30)					
Zinzani *et al;* 1997	Aggressive NHL	N= 149<br>Filgrastim: n = 77<br>No filgrastim: n= 72	-	NA	Grade 4 neutropenia incidence 23.0% vs 55.5%<br>P = 0.00005	Infections 4/77 pts (5%) vs 15/72 pts (21%)<br>P = 0.004<br>Antibiotic use<br>For filgrastim, 4 pts with minor infections required symptomatic treatments and/or oral antibiotics vs<br>For control, 5 pts with major infections and 10 pts with minor infections required parenteral antibiotics and/or hospitalization	NA	Average RDI 95% vs 85%<br>NS	OS at 30 months 64% vs 62%	Musculoskeletal pain 2 (3%) vs 0 (0%)

Ottmann *et al*; 1995	ALL	N= 76<br>Filgrastim: n = 37<br>No filgrastim: n= 39	-	Duration of prolonged neutropenia incidence 22% vs. 42%	NA	Infections<br>43% vs. 56%<br>P = 0.25<br><br>Non viral infections 16 episodes vs. 32 episodes	NA	NA	Death 0 vs. 1	Thrombocytopenia 65% and 58%<br>Fever 35% vs. 47%<br>Musculoskeletal pain 5 patients vs. 7 patients
Maher *et al*; 1994	ALL/Lymphoma	N= 216<br>Filgrastim: n = 109<br>Placebo: n= 107	-	NA	NA	NA	NA	NA	Deaths 11% vs. 14%	Thrombocytopenia 53% vs. 52%<br>Fever mean days 4.1 (0-18) vs. 5.1 (0-28)<br>Musculoskeletal pain 32% vs. 22%<br>Anemia 57% vs. 60%<br>RBC transfusion 65% vs. 62%<br>Platelet transfusion 35% vs 34%
Gebbia *et al*; 1993	Breast Cancer/SCLC /HNC/HC/GC	N= 86<br>Filgrastim: n = 43<br>Placebo: n= 43	ANC 18% vs 42%<br>P <0.05<br>Duration of neutropenia 4.8 days vs. 8.2 days<br>P <0.05	Incidence of FN 12% vs. 32%<br>P <0.05	Grade 3 and 4 neutropenia 18% vs. 42%<br>P <0.05	Oral fungal infection<br>9% vs 21%	NA	RDI 91% vs 71%<br>P <0.05	NA	Muscular and bone pain 5% patients<br>Confusion 2 patients
Trillet-Lenoir *et al*; 1993	SCLC	N= 129<br>Filgrastim: n = 65<br>Placebo: n= 64	-	FN incidence 26% vs 53%<br>P = 0.002	Median duration (days) of neutropenia over 6 cycles:<br>6 vs 15	Infection rate 20% vs 33%<br>P = 0.101<br>Infection-related deaths 1 vs 3<br>IV antibiotics use<br>37% vs 58%<br>P < 0.02	Infection-related hospitalization<br>39% vs 58%<br>P < 0.04	Dose reduction ≥15% over all cycle 29% vs 61%<br>P < 0.001<br>Dose delay ≥2 days in ≥1 cycles 29% vs 47%	Median survival (months)<br>Extensive disease<br>8.9 vs 9.5<br>Limited disease<br>13.9 vs 12.8	Incidence<br>15% vs 9%<br>Musculoskeletal pain, alopecia, nausea, vomiting, stomatitis, diarrhea

Crawford *et al*; 1991	SCLC	N= 199<br>Filgrastim: n = 95<br>Placebo: n= 104	-	FN incidence in cycle 1<br>28% vs 57%<br>P <0.001<br>FN incidence across 6 cycles<br>40% vs 77%<br>P <0.001<br>Median duration (days) in cycle 1<br>4 vs 5<br>NS	Grade 4 neutropenia incidence in cycle 1<br>84% vs 98%<br>P = 0.001<br>Median duration (days) in cycle 1<br>3 vs 6<br>P <0.001<br>Median duration (days) across 6 cycles 1 vs 6	Infection rate across 6 cycles<br>6.5% vs 13.3%<br>G-CSF vs placebo: 51% reduction/cycle<br><br>Mean days of antibiotic use/cycle<br>1.2 vs 2.3<br>RR (placebo vs filgrastim)<br>1.9, 95% CI: 1.44-2.51	Mean days of hospitalization/cycle)<br>2.3 vs 4.2<br><br>Relative risk (placebo vs filgrastim)<br>1.55, 95% CI: 1.26-1.91	NA	Median OS (months)<br>11.4 vs 12.2	Mild to moderate bone pain 20% vs 0%<br>Mild rashes or Itching 6% vs 6%<br>AE leading to withdrawal request (abdominal pain, diffuse aches and pains, preexisting eczema flare-up) 3 (3%) vs 0 (0%)
Acute Myeloid Leukemia (AML)										
Beksac *et al*; 2011	AML	N= 260<br>Filgrastim: n = 123<br>No Filgrastim: n=137	NA	Duration of fever, days<br>8 (1.0–27) vs 8.5 (0.0–28) P = 0.96	NA	NA	Antibacterial therapy<br>91.6% vs 92.4%<br>P = 0.82<br>Antifungal therapy<br>63.0% vs 61.8%<br>P = 0.85<br>Antiviral therapy:<br>8.4% vs 5.3%<br>P = 0.34	Median duration (range) of hospitalization, days<br>31 (9.0–72.0) vs 35 (3.0–80.0)<br>P = 0.18	Median OS duration (SD), days<br>239 (81) vs 184 (65)<br>P = 0.38<br>3-year OS (SD)<br>31.8% (5.6) vs 25.6% (5.1)	Frequent AEs in both arms: rash, musculoskeletal pain, and fever
Heil *et al*; 2006	AML	N= 521	NA	NA	NA	NA	NA	NA	3-year OS (95% CI)	NA

		Filgrastim: n = 259<br>Placebo: n= 262							23% (18–29) vs 21% (16–26)<br>5 year OS (95% CI) 19 (15–24) vs 17 (12–22)	
Usuki *et al*; 2002	AML	N= 245<br>Filgrastim: n = 120<br>No Filgrastim: n=125	Median (95% CI) time to ANC recovery to 1x10⁹/L, days<br>14 (13.9–16.0) vs 22 (19.7–22.7)<br>P < 0.0001<br>Median (95% CI) time to ANC recovery to 0.5x10⁹/L, days<br>12 (1.7–13.5) vs 18 (17.2–20.1<br>P < 0.0001	Incidence of fever<br>76.7% vs 76.0%<br>P = 1.000<br>Median (range) duration of FN, days<br>3 (3.1–4.4) vs 4 (4.1–5.6)<br>P < 0.0001	NA	Rate of infection<br>83.3% vs 91.2%<br>P = 0.083<br>Median (95% CI) duration of infection, days<br>11 (8.3) vs 13 (14.0)<br>P = 0.2320	Rate of IV antibiotic use<br>81.7% vs 87.2%<br>P = 0.100<br>IV antibiotics use, days (range)<br>16.5 (0–49) vs 17 (0–70)<br>P = 0.7039	NA	Median DFS, months<br>14.0 vs 12.5<br>DFS probability (95% CI) at 5 years:<br>34.5% (23.8–43.7%) vs 33.6% (23.3–43.9%)<br>P = 0.9407<br>Median OS, months<br>20.8 vs 18.8<br>OS probability (95% CI) at 5 years	G-CSF-related:<br>Mild musculoskeletal pain (3 pts), fever (1 pt), severe skin rash (1 pt)<br>G-CSF association unknown:<br>Sweet's disease (1 pt), chest pain (1 pt), generalized pruritus, and skin rash (1 pt)

									42.7% (31.4–52.9) vs 35.6% (25.9–45.2)<br>P = 0.5918	
Harousseau *et al*; 2000	AML	N= 194<br>Filgrastim: n = 100<br>No Filgrastim: n=94	NA	Fever duration (association with neutropenia not specified), days:<br>ICC1:<br>5 (0–23) vs 6 (0–25)<br>P = 0.35<br>ICC2:<br>5 (0–31) vs 6 (0–100)<br>P = 0.70	Grade 4 neutropenia duration, days<br>ICC1:<br>12 (5–45) vs 19 (9–39)<br>P < 0.001<br>ICC2:<br>20 (7–56) vs 28 (10–100)<br>P < 0.001	Documented infections:<br>ICC1:<br>55% vs 66%<br>P = 0.16<br>ICC2:<br>40.5% vs 55.5%<br>P = 0.07<br>Episodes of septicemias:<br>ICC1: 40% vs 48%, P = 0.34<br>ICC2: 25% vs 31%, P = 0.05	Median (range) duration of IV antibiotics, days:<br>ICC1:<br>13 (0–34) vs 15 (0–51)<br>P = 0.02<br>ICC2: 15 (0–47) vs 22 (0–100)<br>P = 0.04	Median (range) time of hospital stay, days:<br>ICC1<br>24 (17–100) vs 27 (16–61)<br>P < 0.001<br>ICC2:<br>29 (19–62) vs 34 (21–100)<br>P < 0.001	Deaths:<br>27 pts (27%) vs 31 pts (33%)<br>2-year OS (SD):<br>64% (6%) vs 63% (6%)	NA
Godwin *et al*; 1998	AML	N= 211<br>Filgrastim: n = 106<br>Placebo: n=105	ANC recovery (time from chemotherapy start until neutrophil count >500 /µl, days:<br>24 (75/104 pts recovered) vs 27 (74/103 pts recovered)	NA	15% (95% CI: 3–27) shorter neutropenia duration with filgrastim<br>P = 0.014<br>No difference in thrombocytopenia	Number of ≥3 culture confirmed infections<br>21% vs 21%<br>P = 0.82 one-tailed	Median (range) days on antibiotics<br>22 (0–128) vs 26 (0–69)<br>P = 0.053 one-tailed	Median (range) length of first hospitalization, days<br>29 (4–155) vs 29 (3–106)<br>Median (range) # of febrile days during the first hospitalization:<br>8 (0–79) vs 10 (0–34)<br>P = 0.091 one-tailed	Median survival (95% CI) months<br>6 (3–8) vs 9 (7–10)<br>P = 0.71<br>RFS, months<br>8 (4-10) vs 9 (7–10)<br>P = 0.38	Bone pain: 1 pt (1%) vs 5 pts (5%)<br>Fatal induction toxicities<br>20% (21/104 pts) vs 19% (20/103 pts)

Heil *et al*; 1997	AML	N= 521<br>Filgrastim: n = 259<br>No Filgrastim: n=262	Time to ANC recovery Kaplan-Meier median (95% CI) days for induction 1<br>20 (19–20) vs 25 (24–27)<br>P = 0.0001	Fever incidence<br>Induction 1<br>91% vs 92%<br>P = 0.50<br>Induction 2<br>80% vs 75)<br>P = 0.47<br>Consolidation 1<br>49% vs 63%<br>P = 0.014	Median (range) duration of neutropenia, days<br>Induction 2:<br>10 (0–38) vs 14 (0–43)<br>P = 0.015<br>Consolidation 1:<br>4 (0–46) vs 11 (0–22)<br>P = 0.0001	Infection rate in induction 1<br>37% vs 36%<br>P = 0.85	Use of antibacterials:<br>Induction 1:<br>95% vs 96%<br>Use of anti-infectives<br>Induction 1:<br>95% vs 96%<br>P = 0.81	Median (range) hospital stay, days<br>Induction therapy:<br>23 (2–104) vs 29 (7–93) P = 0.0001<br>Induction and consolidation:<br>42 (15–140) vs 55 (23–114)<br>P = 0.0001	Median survival (95% CI), months<br>DFS:<br>10.1 (8.2–11.4) vs 9.4 (8.2–11.1)<br>P = 0.99<br>OS:<br>12.5 (10.9–14.4) vs 14.0 (12.2–15.6)<br>P = 0.83<br>Deaths in induction phase:<br>21 pts (8.1%) vs 25 pts (9.5%)	AEs in induction 1<br>Rash: 3% vs 2%<br>Musculoskeletal pain: 2% vs 1%
Moore *et al*; 1997	AML	N= 123<br>Filgrastim: n = 61<br>No Filgrastim: n=62	Median days to recovery (95% CI)<br>ANC ≥500/μL<br>20.5 (19–24) vs<br>31.1 (31–36)<br>P < 0.001	NA	NA	Grade ≥3 infections:<br>58% and 47%<br>vs<br>71% and 75%	NA	Incidence of hospitalization<br>47 pts (85%) vs 56 pts (97%)<br>P = 0.05<br>Duration of hospitalization<br>24 (6-44)<br>and 20 (1-58) vs<br>40 (11-91)<br>and 30 (2-80)	Median survival of pts who received third intensification course, years<br>3.4 vs 2.4<br>Death	NA

			Platelets ≥20000/uL 23.4 (19–31) vs 30.2 (26–38)						3 pts vs 3 pts	

Abbreviations: AE= Adverse Event; ANC= Absolute neutrophil count; AML= Acute Myeloid Leukemia; ALL= Acute lymphoblastic leukemia; BEP/EP= Cisplatin, etoposide, bleomycin; CADO= cyclophosphamide, vincristine and doxorubicin; CHOP= Cyclophosphamide, doxorubicin, vincristine, prednisolone; CNOP= Cyclophosphamide, mitoxantrone, vincristine, prednisolone; DFS= Disease free survival; FEC-D= Fluorouracil, Epirubicin, Cyclophosphamide, and Docetaxel; FN= Febrile Neutropenia; G-CSF= Granulocyte- colony stimulating factor; GCM= Germ cell malignancy; IQR= Inter-quartile range; NA= Not available; NHL= Non-Hodgkin Lymphoma; NSCLC= Non-small cell lung cancer; OR= Odds Ratio; OS: overall survival; PP= Primary Prophylaxis; RDI= Relative dose intensity; SCLC= Small-cell lung carcinoma; SD= Standard Deviation.

Table S12. Key characteristics of studies that compared Filgrastim with Pegfilgrastim by indication and study type.

			Efficacy and Effectiveness						Safety	
**Author; Year**	**Cancer Type**	**Patients (N)**	**Time to ANC or platelet recovery**	**Incidence/ Duration of FN**	**Incidence/ Duration of grade 3 or 4 neutropenia**	**Incidence of infection/ antibiotic use**	**Incidence/ Duration of Hospitalization**	**RDI, Dose Delays or dose reductions**	**Survival/ mortality**	**AE**
Chemotherapy-Induced Neutropenia (CIN)										
Fengrui *et al*; 2019	Breast Cancer	N= 339<br>Filgrastim: n = 113<br>MPEG 110 μg/kg: n= 113<br>MPEG 6mg: n= 113	Baseline ANC (±SD)<br>4.21±1.70 in mecapegfilgrastim 100 μg/kg group, 4.18±1.57 in mecapegfilgrastim 6 mg group, and 4.17±1.72 in the filgrastim group	Incidence of FN 2% vs. 5% vs. 0 % (filgrastim vs. mecapegfilgrastim 100 μg/kg vs. mecapegfilgrastim 6 mg/kg)	Mean duration of grade ≥ 3 neutropenia in cycle 1<br>1.06 days in mecapegfilgrastim 100 μg/kg group, 1.23 days in mecapegfilgrastim 6 mg group, and 2.06 days in the filgrastim group<br><br>Incidence of grade ≥3 and grade 4 neutropenia in cycle 1 66.36% vs. 50.45% vs. 50.91% (filgrastim vs. mecapegfilgrastim 100 μg/kg vs. mecapegfilgrastim 6 mg/kg)	NA	NA	NA	NA	In total 98.18% vs. 94.5% vs. 97.2% patient experienced AEs<br><br>Thrombocytopenia: 5% vs. 5% vs. 12%<br>P >0.05<br><br>Back Pain 1% vs. 3% vs. 1%<br>P >0.05<br>Fatigue 9% vs. 16% vs. 14%<br>P >0.05<br>Muscle pain 1% vs. 7% vs. 6%<br>P >0.05
Kubo *et al*; 2016	NHL or HL	N= 111<br>Filgrastim: n = 56<br>Pegfilgrastim: n= 55	-	Incidence of FN 55.6% vs. 56.6%	Mean duration of severe neutropenia was 4.7 days vs. 4.5 days<br>P < 0.001	NA	NA	NA	NA	Vomiting 2% vs. 15%<br>Back Pain 29.1% vs. 22.2%<br>Bone Pain 9.1% vs. 0%<br>Platelet count decreased

										100% vs. 98.1% Pyrexia 25.5% vs. 22.2%
Park *et al*; 2016	Breast Cancer	N= 74 Filgrastim: n = 38 DA 3031: n= 36	-	Incidence of FN 7.9% vs. 17.1%	Mean duration of Grade 4 neutropenia in cycle 1 2.08 ± 0.85 days for the filgrastim group and 2.28 ± 1.14 days for the DA-3031 group	NA	Incidence of hospitalization 36.8% vs. 37.1% P = 0.9788	NA	NA	Severe AEs reported in both groups were 94.7% vs. 97.2% Serious AEs 15.8% vs. 27.8%
Filon *et al*; 2015	Breast Cancer	N= 135 Filgrastim: n = 45 EPEG 6 mg/kg: n= 45 EPEG 7.5 mg: n= 45	-	Incidence of FN in all cycles 2.5% vs. 2.38% vs. 6.98% Incidence of severe neutropenia in all cycles 100% vs. 95.24% vs. 79.07%	Mean duration of grade 4 neutropenia was 1,725 days vs. 0,905 days vs. 0,791 days	NA	NA	NA	NA	myalgia 7.14% vs. 4.65% vs. 4.65%, arthralgia 14.29% vs. 6.98% vs. 6.98%, ossalgia 9.52% vs. 9.30% vs. 4.65%, local reactions 7.14% vs. 2.33% vs. 2.33%)
Zhang *et al*; 2015	Breast Cancer	N= 171 Filgrastim: n = 43 PEG 60 μg /kg: n= 43 PEG 100 μg/kg: n= 43 PEG 120 μg/kg: n= 42	Time to ANC recovery 1.26 days vs. 1.49 days vs. 1.16 days vs. 1.24 days	Incidence of FN 11.63% vs. 6.98% vs. 4.65% vs. 11.90%	Mean duration of grade 3+ neutropenia 1.69 days vs. 2.09 days vs. 1.53 days vs 1.73 days P = 0.043	NA	NA	NA	NA	AEs in total 90.70% vs. 95.35% vs. 90.70% vs. 92.86% Bone Pain 16.28% vs. 4.65% vs. 4.65% vs. 9.52%
Park *et al*; 2013	Breast Cancer	N= 61 Filgrastim: n = 21	Time to ANC recovery 9.8±0.8 vs. 10.1± 1.8 vs. 9.9±1.6	Incidence of FN 9.5% vs. 15% vs. 5%	Mean duration of G4 neutropenia 2.48±1.03 days vs. 2.20±1.47 days vs. 2.05±1.05 days	NA	NA	NA	NA	Musculoskeletal and connective tissue disorders

		DA-3031 3.6 mg: n= 20<br>DA-3031 6 mg : n= 20			P= 0.275					28.6% vs. 21.1% vs. 38.1%<br>P = 0.495<br><br>Back Pain 14.3% vs. 28.6% (DA-3031 6 mg)<br><br>Nervous system disorder 9.5% vs. 5.3% (DA-3031 6 mg)<br>P= 0.522
Salafet *et al*; 2013	Breast Cancer	N= 60<br>Filgrastim: n = 19<br>BCD 017 3 mg: n= 21<br>BCD 017 6 mg: n= 20	-	Incidence of severe neutropenia in 1st cycle<br>61.1% vs. 85.7% vs. 65%	Mean duration of grade 4 neutropenia in cycle 1 was 0.33 days vs. 0.43 vs. 0.40	NA	NA	NA	NA	NA
Green *et al*; 2003	Breast Cancer	N= 152<br>Filgrastim : 75<br>Pegfilgrastim: 77	Time to ANC recovery 9 days for both group	Incidence of FN 15% vs 9%	Mean duration of grade 4 neutropenia in cycle 1 was 1.6 days vs 1.8 days	i.v. antibiotic administration 21% and 17%	Hospitalization 31% and 18%	NA	NA	58% vs 57% patients experienced one of AE<br>Bone pain 42% vs 37%<br>grade 4 anemia 4% vs 0% and grade 4 thrombocytopenia 1% vs 0%
Grigg *et al*; 2003	NHL	N= 50<br>Filgrastim : 22<br>Pegfilgrastim: 27		Incidence of FN in cycle 1 was 0% vs 15%	Mean duration of grade 4 neutropenia in cycle 1 was 0.8 days vs 1.5 days	NA	NA	NA	NA	Adverse events (WHO grade 1–4) were reported by 95%

										of filgrastim and 96% of pegfilgrastim patients.<br>Bone pain 50% vs 30%
Vose *et al*; 2003	Relapsed or refractory HL or NHL	N= 60<br>Filgrastim : 31<br>Pegfilgrastim: 29	Mean ANC nadir was 0.208 x 10^9/L vs. 0.161 x 10^9/L	Incidence of FN 19% vs. 21%	Incidence of grade 4 neutropenia 68% vs. 69%<br><br>Mean duration of grade 4 neutropenia 2.4 days vs. 2.8 days	NA	NA	NA	Deaths overall 5 vs. 3	Fatigue 19% vs. 14%<br>Thrombocytopenia 6% vs. 14%<br>Anemia 13% vs. 3%<br>Fever 10% vs. 3%<br>Granulocytopenia 13% vs. 3%
Holmes *et al*; 2002	Breast Cancer	N= 296<br>Filgrastim : 147<br>Pegfilgrastim: 149	Mean time to ANC recovery 9.7 days vs. 9.3 days	Incidence of FN overall<br>18% vs. 9%	Incidence of grade 4 neutropenia in cycle 1<br>79% vs. 77%<br><br>Mean duration of grade 4 neutropenia 1.8 days vs. 1.7 days	NA	NA	NA	NA	Skeletal pain 26% vs. 25%<br>Serious AEs 20% vs. 19%
Holmes *et al*; 2002	Breast Cancer	N= 125<br>Filgrastim: n= 25<br>PEG 30 µg /kg: n= 19<br>PEG 60 µg/kg: n= 60<br>PEG 100 µg/kg: n= 46	Mean time to ANC recovery 9.4 days vs. 11 days vs. 10.3 days vs. 9.5 days	Incidence of FN 4% vs. 21% vs. 8% vs. 7%	Incidence of grade 4 neutropenia 76% vs. 95% vs. 90% vs. 74%<br><br>Mean duration of grade 4 neutropenia 1.6 days vs. 2.7 days vs. 2 days vs. 1.3 days	NA	NA	NA	NA	Bone Pain 36% vs. 35%
Acute Myeloid Leukemia (AML)										
Sierra *et al*; 2008	AML	N= 83<br>Filgrastim: n= 41<br>Pegfilgrastim: n= 42	Median time to ANC recovery was 16.5 days vs. 17.0 days	Incidence of FN 88% vs. 81%	Fever 58% vs77%	NA	NA	NA	Deaths 2 vs 1	Bone pain 10% vs 7%

Table S13. Key characteristics of studies that compared Filgrastim with Biosimilar Filgrastim by indication and study type.

			Efficacy and Effectiveness						Safety	
**Author; Year**	**Cancer Type**	**Patients (N)**	**Time to ANC or Platelet Recovery**	**Incidence/ Duration of FN**	**Incidence/ Duration of Grade 3 or 4 Neutropenia**	**Incidence of Infection/ Antibiotic Use**	**Incidence/ Duration of Hospitalization**	**RDI, Dose Delays or Dose Reductions**	**Survival/ mortality**	**AE**
Chemotherapy-Induced Neutropenia (CIN)										
Blackwell *et al*; 2018	Breast Cancer	N= 213<br>Filgrastim: n = 51<br>Switched biosimilar: n= 109<br>EP2006: n= 53	-	Incidence of FN across cycles 2-6[d]<br>0% vs. 3.4%	NA	Infections 9.9% vs. 9.3%	Hospitalization 0 vs. 1	NA	NA	Treatment related AEs 39.2% vs. 42.1%<br>Musculoskeletal/connective tissue Disorders 39.2% vs. 35.5%<br><br>Bone pain 33.3% vs. 30.8%
Hegg *et al*; 2016	Breast Cancer	N= 217<br>Filgrastim: n= 108<br>Biosimilar filgrastim: n= 109	-	Rate of FN<br>2.38% vs. 3.49%<br>P= 0.669	Patients with Grade 4 neutropenia 54.6 % vs. 51.4%<br>P= 0.6311	NA	NA	NA	NA	Mild to moderate AEs 105 pt. vs. 101pts.<br><br>Serious AE<br>6 pt. vs. 3 pt.

Blackwell *et al*; 2015	Breast Cancer	N= 214<br>Filgrastim: n= 107<br>EP 2006: n= 107	Time to ANC recovery (median) was reference (range: 0−4) and 2.0 days for biosimilar (range: 0−6)	NA	Fever 2.8% vs. 6.6%<br><br>Grade ¾ neutropenia 79% vs. 77%	NA	NA	NA	NA	Treatment related AEs 19.6% vs. 20.6%
Waller *et al*; 2010	Breast Cancer	N= 278<br>Filgrastim: n= 95<br>Biosimilar filgrastim: n= 183	-	Incidence of FN in cycle 1-3<br>2.4% vs. 2.4%	Incidence of severe neutropenia in cycle 1<br>68.2% vs. 77.6%	Infections 3.5% vs. 3%	NA	NA	NA	In both treatment groups, the most common treatment-emergent AEs of any grade were nausea, fatigue, and bone pain.<br>Any AE 84.2% vs. 86.9%<br>Bone Pain 16.8% vs. 26.2%<br>Myalgia 9.5% vs. 14.2%
del Giglio *et al*; 2008	Breast Cancer	N= 348<br>Filgrastim: n = 136<br>XM02: n= 140<br>Placebo/<br>XM02: n= 72	-	FN incidence 20.7% vs 22.1% vs 41.7%	Mean duration (days) of severe neutropenia<br>Cycle 1<br>1.1 vs 1.1 vs 3.8<br>Cycle 4<br>0.7 vs 0.7 vs 0.6	NA	NA	NA	3 deaths in cycle 1<br>1 sepsis and 1 cardiorespiratory arrest in placebo; 1 ischemic stroke in XM02	Most commonly reported drug-related AEs<br>bone pain (10.3%)<br>asthenia (7.8%)<br>myalgia (6.3%)<br>diarrhea (5.2%)

			Efficacy and Effectiveness						Safety	
**Author; Year**	**Cancer Type**	**Patients (N)**	**Time to ANC or platelet recovery**	**Incidence/ Duration of FN**	**Incidence/ Duration of grade 3 or 4 neutropenia**	**Incidence of infection/ antibiotic use**	**Incidence/ Duration of Hospitalization**	**RDI, Dose Delays or dose reductions**	**Survival/ mortality**	**AE**
Chemotherapy-Induced Neutropenia (CIN)										
Blackwell *et al*; 2018	Breast Cancer	N= 213 Filgrastim: n = 51 Switched biosimilar: n= 109 EP2006: n= 53	-	Incidence of FN across cycles 2-6[d] 0% vs. 3.4%	NA	Infections 9.9% vs. 9.3%	Hospitalization 0 vs. 1	NA	NA	Treatment related AEs 39.2% vs. 42.1% Musculoskeletal/connective tissue Disorders 39.2% vs. 35.5% Bone pain 33.3% vs. 30.8%
Hegg *et al*; 2016	Breast Cancer	N= 217 Filgrastim: n= 108 Biosimilar filgrastim: n= 109	-	Rate of FN 2.38% vs. 3.49% P= 0.669	Patients with Grade 4 neutropenia 54.6 % vs. 51.4% P= 0.6311	NA	NA	NA	NA	Mild to moderate AEs 105 pt. vs. 101pts. Serious AE 6 pt. vs. 3 pt.

Blackwell *et al*; 2015	Breast Cancer	N= 214<br>Filgrastim: n= 107<br>EP 2006: n= 107	Time to ANC recovery (median) was reference (range: 0−4) and 2.0 days for biosimilar (range: 0−6)	NA	Fever 2.8% vs. 6.6%<br><br>Grade ¾ neutropenia 79% vs. 77%	NA	NA	NA	NA	Treatment related AEs 19.6% vs. 20.6%
Waller *et al*; 2010	Breast Cancer	N= 278<br>Filgrastim: n= 95<br>Biosimilar filgrastim: n= 183	-	Incidence of FN in cycle 1-3<br>2.4% vs. 2.4%	Incidence of severe neutropenia in cycle 1<br>68.2% vs. 77.6%	Infections 3.5% vs. 3%	NA	NA	NA	In both treatment groups, the most common treatment-emergent AEs of any grade were nausea, fatigue, and bone pain.<br>Any AE 84.2% vs. 86.9%<br>Bone Pain 16.8% vs. 26.2%<br>Myalgia 9.5% vs. 14.2%
del Giglio *et al*; 2008	Breast Cancer	N= 348<br>Filgrastim: n = 136<br>XM02: n= 140<br>Placebo/<br>XM02: n= 72	-	FN incidence 20.7% vs 22.1% vs 41.7%	Mean duration (days) of severe neutropenia<br>Cycle 1<br>1.1 vs 1.1 vs 3.8<br>Cycle 4<br>0.7 vs 0.7 vs 0.6	NA	NA	NA	3 deaths in cycle 1<br>1 sepsis and 1 cardiorespiratory arrest in placebo; 1 ischemic stroke in XM02	Most commonly reported drug-related AEs<br>bone pain (10.3%)<br>asthenia (7.8%)<br>myalgia (6.3%)<br>diarrhea (5.2%)

Studies	Random sequence generation (selection bias)	Allocation concealment (selection bias)	Blinding of participants and personnel (performance bias)	Blinding of outcome assessment (detection bias)	Incomplete outcome data (attrition bias)	Selective reporting (reporting bias)	Other bias
Crawford et al; 2005	−	?	+	+	+	+	+
Timmer-Bonte et al; 2005	?	?	−	?	+	+	+
Doorduijn et al; 2003	?	?	?	?	+	+	+
Osby et al; 2003	?	?	?	?	+	+	+
Papaldo et al; 2003	?	?	?	−	+	?	+
Fossa et al; 1998	?	?	?	?	+	+	+
Larson et al; 1998	+	?	+	?	+	+	+
Michon et al; 1998	+	?	−	?	+	+	+
Geissler et al; 1997	?	?	?	?	+	+	+
Pui et al; 1997	?	?	?	?	+	+	+
Zinzani et al; 1997	?	?	?	?	+	+	+
Maher et al; 1994	+	?	+	+	+	+	+
Trillet-Lenoir et al; 1993	?	?	+	+	+	+	+
Crawford et al; 1991	−	?	+	+	+	+	+
Beksac et al; 2011	+	+	?	−	+	+	+
Heil et al; 2006	?	?	+	?	+	+	+
Usuki *et al*; 2002	−	?	?	?	+	+	+
Harousseau *et al*; 2000	−	?	?	?	+	+	+
Godwin *et al*; 1998	−	?	+	+	+	+	+
Heil *et al*; 1997	?	?	+	?	+	+	+
Dale et al; 1993	+	?	+	+	+	+	+
Gonzalez-Vicent et al; 2004	+	?	+	?	+	+	+
Stahel et al; 1997	?	?	+	?	+	+	+
Fengrui *et al*; 2019	−	?	+	+	+	+	+
Kubo *et al*; 2016	+	?	+	?	+	+	+
Park *et al*; 2017	−	?	−	?	+	+	+
Filon *et al*; 2015	+	?	+	+	+	+	+
Zhang *et al*; 2015	+	?	?	?	+	+	+
Park *et al*; 2013	+	?	−	?	+	+	+
Salafet *et al*; 2013	+	?	−	?	+	+	+
Green *et al*; 2003	+	+	+	?	+	+	+
Grigg *et al*; 2003	?	?	−	?	+	+	+
Vose *et al*; 2003	?	?	−	?	+	+	+
Holmes *et al*; 2002	?	?	+	+	+	+	+
Holmes *et al*; 2002	?	?	+	+	+	+	+
Sierra et al; 2008	+	+	+	+	+	+	+
Blackwell *et al*; 2018	?	+	+	+	+	+	+
Blackwell *et al*; 2015	+	+	+	+	+	+	+
Hegg *et al*; 2016	?	?	−	?	+	+	+
Waller *et al*; 2010	+	?	+	+	+	+	+
Engert *et al*; 2009	?	+	−	+	+	+	+
del Giglio *et al*; 2008	?	+	−	+	+	+	+

Figure S1. Cochrane Collaboration Risk of Bias Assessment Tool version 2 (RoB 2).

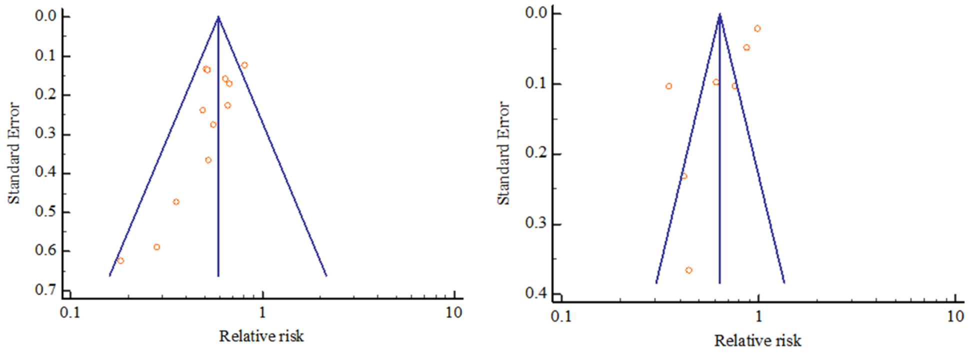

(a). (b)

Figure S2. Funnel Plots for Filgrastim versus Placebo/ No treatment. (**a**). Febrile Neutropenia; (**b**) Grade 3 or 4 Neutropenia.

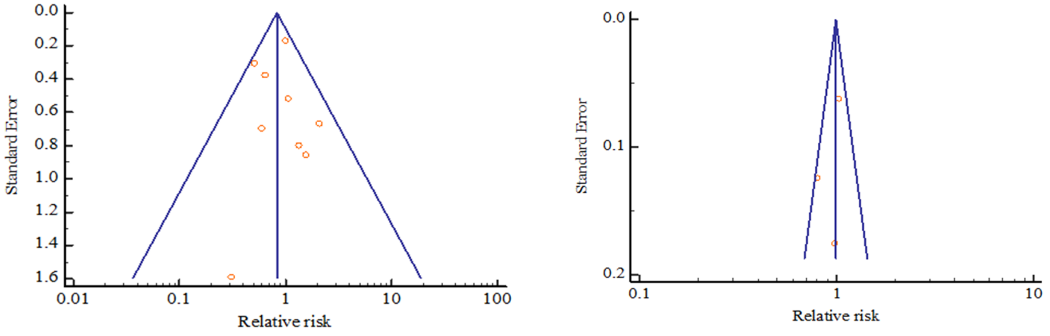

(a) (b)

Figure S3. Funnel Plots for Pegfilgrastim versus Filgrastim. (**a**). Febrile Neutropenia; (**b**) Grade 3 or 4 Neutropenia.
